# Evaluation of Damage Stress Thresholds and Mechanical Properties of Granite: New Insights from Digital Image Correlation and GB-FDEM

**DOI:** 10.1007/s00603-024-03789-7

**Published:** 2024-03-06

**Authors:** Kareem Ramzy Aboayanah, Aly Abdelaziz, Bezawit Fekadu Haile, Qi Zhao, Giovanni Grasselli

**Affiliations:** 1 Department of Civil and Mineral Engineering, University of Toronto, 35 St George St., Toronto, ON M5S 1A4 Canada; 2 Department of Civil and Environmental Engineering, The Hong Kong Polytechnic University, Hung Hom, Hong Kong SAR, China

**Keywords:** Grain-based modeling, Digital image correlation, Crack opening, Damage stress thresholds, Grain size effect, FDEM

## Abstract

We employed a novel combination of digital image correlation (DIC) and grain-based hybrid finite–discrete element method (GB-FDEM) to improve the comprehension of the relationships between microstructural features and the mechanical properties of granitic rocks. DIC and numerical results showed that macrocracks initiated and propagated along grain boundaries among different minerals driven by the high stiffness contrast between the compliant biotite and the stiffer feldspar/quartz grains. Surface deformation analyses revealed that tensile-dominated macrocracks open at monotonically increased rates before the crack damage threshold, and the opening accelerated afterwards with the increased shear component. The onset of the acceleration of the opening rate of macrocracks can be used to infer the crack damage threshold. Both strain and acoustic emission were used to infer damage stress thresholds in the synthetic numerical samples. Numerical results showed that the damage stress thresholds and uniaxial compressive strength decrease with increasing grain size following log-linear relations. Coarse-grained samples tend to fail by axial splitting, while fine-grained samples fail by shear zone formation. Biotite and quartz contents significantly affect mechanical properties, while quartz to feldspar ratio is positively related to the mechanical properties. Our study demonstrates the capacities of DIC and GB-FDEM in inferring damage conditions in granitic rocks and clarifies the microstructural control of the macroscopic mechanical behaviors. Our results also provide a comprehensive understanding of the systematics of strain localization, crack development, and acoustic emission during the rock progressive failure process.

**Supplementary Information:**

The online version contains supplementary material available at 10.1007/s00603-024-03789-7.

## Highlights

- Digital image correlation analysis and numerical modeling using grain-based hybrid finite–discrete element method can be used to evaluate damage stress thresholds.
- The onset of the acceleration of the crack opening can be used to infer the crack damage threshold.
- Strain field from digital image correlation revealed that stiffness contrast between mineral grains act as nuclei for crack initiation.
- Damage stress thresholds and uniaxial compressive strength decrease with increasing grain size following log-linear relations.

**Supplementary Information:**

The online version contains supplementary material available at 10.1007/s00603-024-03789-7.

## Introduction

Rocks are heterogeneous materials with mechanical properties that largely vary according to morphology, stress state, degree of intactness, and mineralogy. Their heterogeneity can explain the variations in tensile and compressive strength exhibited by consecutive samples collected and tested from the same core, even after the experimental system compliance is taken into consideration (Abdelaziz and Grasselli 2021). Granite is an example of such heterogeneous crystalline rock comprising mainly quartz, feldspar, and biotite. Understanding and correlating rock microstructural features and mineralogy to its mechanical properties have always captivated rock mechanics researchers. Using experimental studies that combine traditional laboratory compressive strength tests with advanced petrographic image analysis and acoustic emission (AE), researchers were able to investigate the effect, and determine the impact of mineralogical compositions and morphologies on the mechanical strength of the rocks (Eberhardt et al. 1999; Přikryl 2001; Sun et al. 2017). Several studies report an increase in uniaxial compressive strength (UCS) of granitic rocks with the decrease in grain size (Brace 1961; Mendes et al. 1966; Tahir et al. 2019; Tuğrul and Zarif 1999; Yilmaz et al. 2009). Tuğrul and Zarif (1999) reported higher strength in samples with higher quartz to feldspar ratio, while Yilmaz et al. (2009) did not observe any significant effect of quartz content on the UCS. Mineral cleavage planes, microcracks, and grain boundaries were noticed to also have significant impacts on rock strength and failure pattern (Tullis and Yund 1977; Willard and McWilliams 1969). Přikryl (2001) investigated microstructural features including grain size, shape, and orientation on mechanical parameters of different crystalline rock types by combining compressive tests and thin sections petrographic analysis and reported a log-linear correlation between mean grain size and the UCS. In addition, crack initiation stress (CI) and its dependence on mineralogy and microstructure were also investigated. Nicksiar and Martin (2013) analyzed a dataset of more than 300 rock samples and showed that a higher CI threshold is observed in fine-grained and quartz-rich igneous rocks, contradicting earlier observation by Eberhardt et al. (1999) that reported an insignificant effect of grain size on the crack initiation stresses. However, it was observed that once cracks initiate, the grain size has a significant effect on their propagation (Eberhardt et al. 1999).

Various mechanisms including stress concentration at extensional crack tips, sliding at grain boundaries, pre-existing cracks activation, stress concentration at indenting grains contacts, and stiffness contrast between different grains are responsible for crack initiation in rocks under stresses (Brace et al. 1966; Tapponnier and Brace 1976). Experimental observations have revealed that the number of stress-induced microcracks increases with the stress level experienced by the sample (Brace et al. 1966; Ghasemi et al. 2020; Tapponnier and Brace 1976). Numerical studies have also captured the increase in microcracks formation with increased stress (Abdelaziz et al. 2018; Hofmann et al. 2015; Peng et al. 2017). Microcracks in crystalline rocks can be categorized into three groups: grain boundary cracks (i.e., intergranular crack) forming along grains boundaries, intragranular cracks lying entirely within the grains, and transgranular cracks which crosses grain boundaries into the grain itself (Kranz 1983). In granite, transgranular cracks are observed propagating from biotite grain boundaries due to the stiffness contrast between biotite and stiffer minerals such as quartz and feldspar (Tapponnier and Brace 1976). This highlights the critical role of biotite, a mineral with weak cleavage planes, in influencing the fracturing process in granites. Studies on intergranular and transgranular cracks in granite under tension documented the effects of the anisotropic nature of biotite and feldspar, as cleavage grains, on crack orientation (Kudo et al. 1992). They also observed that without pre-existing cracks in the granite, cracks are hard to initiate in quartz due to its higher strength compared to the other minerals. Studies were also carried out to investigate crack types and their evolution in the major minerals constituting granite as well as the relation to grain shape through AE and linear microcrack density calculation from petrographic thin sections (Ghasemi et al. 2020).

Numerically, various computational methods have been employed to model and investigate the behavior of rocks. Continuum approaches such as the finite-difference method (FDM) and the finite-element method (FEM) have been extensively used to study fracture nucleation in brittle materials (Areias et al. 2018; Bordas et al. 2011; Linder and Raina 2013; Rinaldi et al. 2015; Sharafisafa and Nazem 2014; Sukumar et al. 2000; Verma and Singh 2010). However, these approaches are unable to capture large deformations such as slipping and spalling associated with brittle deformation of geomaterials. Discontinuum approaches have been used to capture such behaviors. Distinct element method (DEM) (Hart and Cundall 1992) mainly adopts two approaches for studying the fracturing in brittle materials: polygon-based DEM (Li et al. 2017; Zhou et al. 2017) and particle-based DEM (Hazzard and Young 2000; He et al. 2023; Potyondy and Cundall 2004). Although the latter provides more flexibility in capturing fracture propagation, both methods use non-fracturable rigid elements with pre-determined geometries. The hybrid finite–discrete element method (FDEM), which combines capabilities of continuum and discontinuum approaches (Munjiza 2004), has been successfully adopted to investigate the microstructure and fracture propagation in rocks in models ranging from laboratory testing to field scale applications (Abdelaziz et al. 2018; Aboayanah et al. 2023; He et al. 2018; Mahabadi et al. 2012; Paluszny et al. 2016; Seyed Ghafouri et al. 2022; Shao et al. 2022; Sun et al. 2022a, b, 2023; Zhao et al. 2015; Zhou et al. 2020). Using this approach, Lisjak et al. (2013) and Zhao et al. (2015) modeled and successfully captured AEs generated by crack growth using a quasi-dynamic approach.

Recently, the grain-based modeling (GBM) approach, which allows for a more realistic representation of rock microstructure by dividing the model domain into cells mimicking mineral crystals in the rock, has been adopted to investigate microcracking patterns at the grain scale. GBM was implemented in PFC2D to study the impacts of grain size and grain size heterogeneity on mechanical properties and cracking patterns in crystalline rocks (He et al. 2023; Hofmann et al. 2015; Peng et al. 2017; Potyondy 2010; Saadat and Taheri 2019a, b). GBM-PFC was also adopted to numerically investigate the impacts of grain size and pre-existing fractures on the mechanical properties of granitic rocks (Hofmann et al. 2015; Saadat and Taheri 2019a, b). Curved surfaces provided by the particles concentrate stresses only at points of contact, and hence, it is not suitable for modeling angular grains. Other researchers have implemented GBM in the polygon-based Universal Discrete Element Code (UDEC) to investigate the relationship between grain size and mechanical properties (Gao et al. 2016; Huang et al. 2021; Li et al. 2017; Wang and Cai 2019). Despite the fact that the majority of polygon-based models are limited to rigid polygons thus inhibiting the formation of intragranular cracks (Gao et al. 2016), intragranular cracking was captured in the polygon-based UDEC using Mohr–Coulomb strain-softening constitutive model assigned to different mineral blocks (Sinha et al. 2020). The polygon-based fully fracturable mesh used in FDEM method resolves such an issue (Abdelaziz et al. 2018; Aboayanah et al. 2021; Li et al. 2021).

Intergranular and intragranular cracks evolution in Stanstead granite was modeled using GBM approach implemented in FDEM (Abdelaziz et al. 2018). Following a similar approach, the effects of boundary conditions, slenderness, and loading rate were studied on granite strength and cracking patterns (Li et al. 2020). The influence of grain size and the presence of pre-existing cracks was also studied using grain-based FDEM (Abdelaziz et al. 2018; Li et al. 2021), and the particle growth approach was implemented in Voronoi grain-based FDEM to model crystalline rock microstructure in 3D (Zhou et al. 2020). Recently, the thermal cracking of Stanstead granite was simulated using a thermo-mechanical grain-based FDEM approach (Aboayanah et al. 2022, 2021). Therefore, by utilizing GBM to capture the microstructure of granitic rocks and combining it with FDEM methods, this study will investigate the effect of grain size and mineralogy on the mechanical properties and microcracking patterns.

Digital image correlation (DIC), which is an experimental approach based on tracking the deformation of the object surface, has been used to study the mechanical behavior of rocks (Dong et al. 2017; Dutler et al. 2018; Lin and Labuz 2013; Lin et al. 2014; Wu et al. 2020). In this paper, we exploited the capabilities of this method to infer new insights on the evolution of stress damage thresholds, cracks opening, and failure mode during the uniaxial loading of granitic rocks. We have also supplemented the DIC with FDEM to investigate the effects of microstructures on damage stress thresholds and the mechanical behavior of granitic rocks.

This paper is structured such that it first explores the implementation of the DIC technique to infer crack damage thresholds and failure patterns during uniaxial compression of granitic rocks. Then, it illustrates the modeling approach in GB-FDEM framework followed by micromechanical parameters calibration using three different granitic rocks with different grain sizes. The calibrated parameters are then used to conduct a parametric analysis on the effects of grain size and mineralogy on UCS, damage stress thresholds, and Young’s modulus (E). The paper then discusses the results and their implications followed by concluding statements.

## Methodology

### Sample Preparation and Testing

Four coarse-grained granitic samples (S-1, S-2, S-3, and S-4) were prepared to be tested under uniaxial compressive conditions. The sample size was 38 mm in diameter and 98 mm in height and their surfaces were ground, polished, and sprayed with black-on-white speckles pattern for the deformation to be tracked using DIC. The tests were carried out on a Forney test frame and complied, where possible, to internationally recognized standards. The UCS tests were carried out in accordance with ASTM D7012-14e1 and adhered, where applicable, to the sample tolerances stipulated in ASTM D4543-19. During the UCS test, the axial strain was recorded using linear variable differential transformers (LVDTs) (LD Sensors LDS-10). The DIC was also used to evaluate the strain occurring on the surface of the tested sample. In all tests, the applied axial load was captured using a pressure transducer (Ashcroft GV 3000 psi) mounted on the test frame and the pressure was regulated through a high-precision/pressure metering pump (Vindum VP-6K). A three-dimensional DIC (two Basler acA2500–60 um cameras in stereo setup) system was used to capture the deformation on one side of the sample. The pressure transducer and LVDTs were connected to a digital acquisition system (NI USB-6001). During the test, the first image captured by the cameras was used as the reference state for calculating the deformations. Image correlation was achieved using GOM Correlate Pro software. To validate the DIC measurement, a 25 mm diameter aluminum 6061-T6 (Al) sample was uniaxially loaded. Axial strain from the DIC was recorded and averaged from two 5 mm axial virtual LVDTs placed on the middle of the sample. The calculated elastic modulus of the aluminum sample from the strain field measured by the DIC is 69.5 GPa which is in agreement with the theoretical elastic modulus in compression of 69.7 GPa (ASM 1990).

DIC can be described as a form of the particle tracking approach, where the displacement vector of a group of particles (called speckles) is tracked (Belrhiti et al. 2012). This group of speckles has a unique intensity pattern and is called the “subset”. In this technique, a digital camera is employed to record digital images of the sample surface sprayed with the speckle pattern and a cross-correlation algorithm is employed to calculate the deformation between the reference and the subsequent images. Larger than the subset, the region of interest (ROI) serves as the area where the subset is traced. For experiments involving small displacements, a tracing approach based on the fast Fourier transform (FFT) and characterized by efficient computation was used. We also developed a post-processing approach in Python (Van Rossum and Drake 2009) to analyze the displacement field evolution to detect cracks and determine damage thresholds. This script detects cracks which appear as discontinuities or jumps on the displacement curves and uses the boundaries of these discontinuities to calculate the opening of the cracks.

### Model Setup

**Fig. 1:**
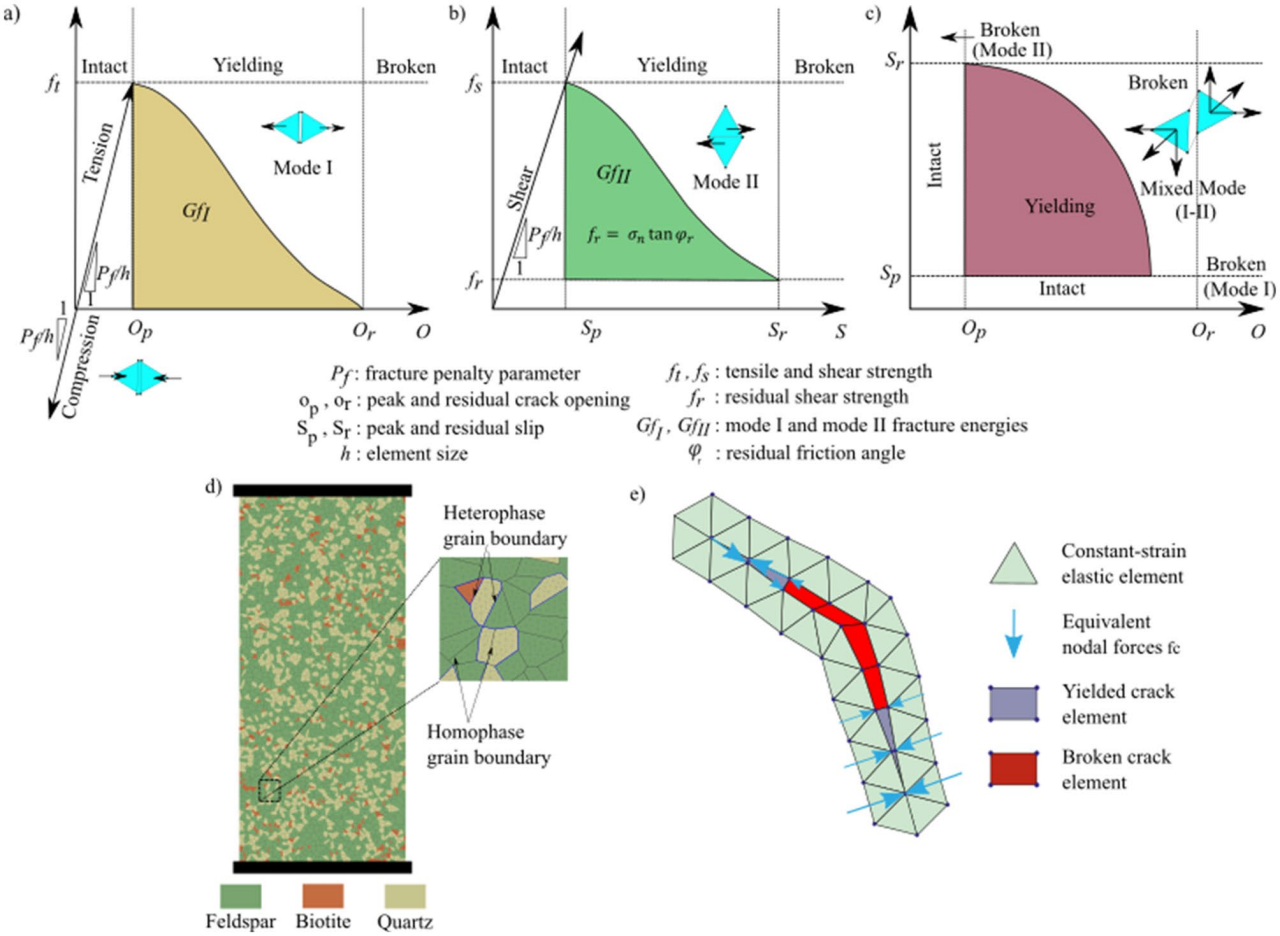
Constitutive laws used in FDEM to model **a** tensile failure (mode I), **b** shear failure (mode II), and **c** mixed-mode failure (mode I & II). **d** Grey granite synthetic sample with an illustration of minerals and grains interfaces. **e** Opening and sliding (mode I and II, respectively) simulation in FDEM

The FDEM combines the DEM that has the capability to capture fracture propagation, slip, and interaction with the FEM that models small deformations and elastic behavior effectively. Fracture initiation and propagation are simulated in FDEM using the principles of non-linear elastic fracture mechanics (Lisjak et al. 2013). In two-dimensional modeling, between any two adjacent triangular FEM elements, there is a four-node cohesive crack element (CCE) to simulate crack propagation based on energy-based failure criteria (Fig. 1). Thus, no prior assumptions for crack trajectory are necessary and any arbitrary fracture trajectory can be captured within the limitations of the mesh topology. The constitutive relations used for fracture simulation in FDEM are illustrated in Fig. 1a-c.

**Fig. 2:**
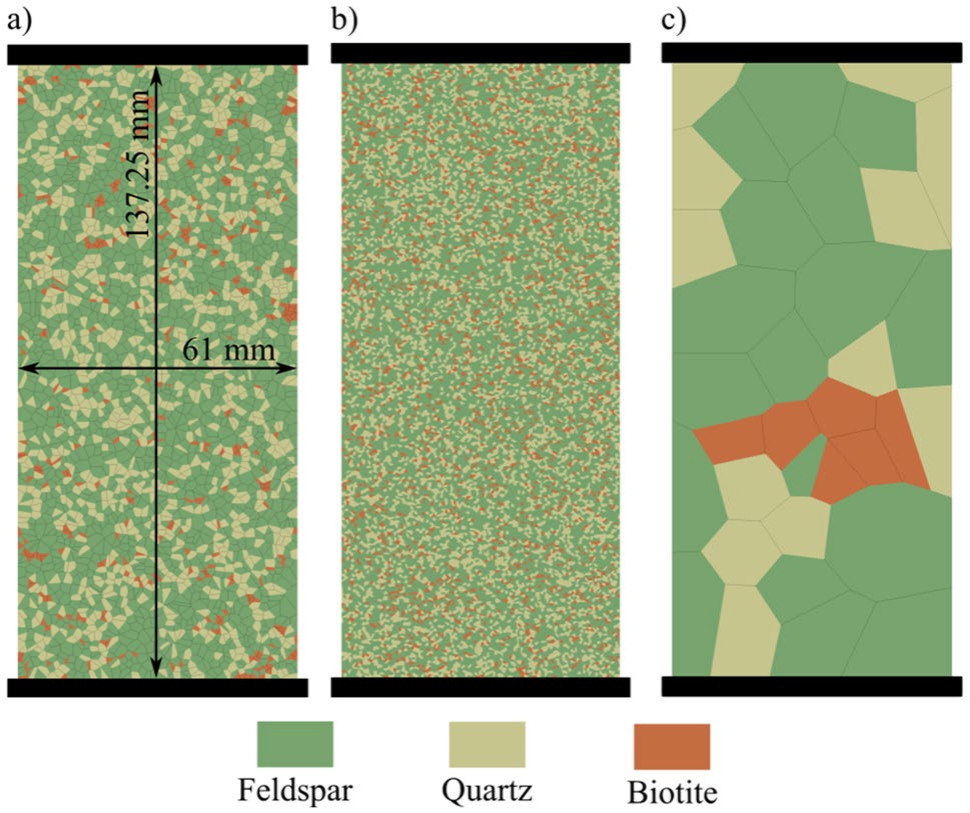
Synthetic granitic samples of size 61 mm by 137.25 mm used in model calibration: **a** grey granite, **b** granodiorite, and **c** pegmatite

We used data from the literature for granitic rocks collected from the same site (the Atomic Energy of Canada Limited’s underground research laboratory) to rigorously capture the effect of grain size and mineral proportions instead of using granites collected from different locations. Experimental data from three different types of granitic rock samples, namely grey granite, granodiorite, and pegmatite were used to calibrate and validate the synthetic samples presented in the study (Diederichs et al. 2004; Eberhardt et al. 1999; Eberhardt 1998). It is worth mentioning that the experimental UCS value for pegmatite was estimated from its crack damage (CD) value considering CD/UCS ratio of 0.78 (Xue et al. 2014). The synthetic rock samples were generated using Voronoi tessellations to produce realistic models of the simulated granitic rocks (Fig. 2). Table 1 details the dimensions, mineral compositions, and grain sizes of the synthetic samples. The mineralogical composition and grain size synthetic distributions adopted resemble actual mineral grain sizes reported in literature (Eberhardt et al. 1999) to realistically represent the studied samples. For this study, the percentages of plagioclase and K-feldspar were combined into a single mineral phase named “feldspar”.

**Table 1:** Synthetic granitic samples used in the study

Model		Feldspar	Biotite	Quartz
Grey granite	Composition (%)	65	5	30
	Grain size (mm)*	0.9–3.9 (3.1)	0.77–1.36 (0.9)	1.36–2.3 (1.9)
Granodiorite	Composition (%)	60	10	30
	Grain size (mm)*	0.72–1.49 (1.0)	0.64–0.72 (0.6)	0.72–0.84 (0.7)
Pegmatite	Composition (%)	61	11	28
	Grain size (mm)*	16–28 (20)	11–13.5 (12.5)	9.8–15.8 (14.4)

*Grain size is reported as Min.–Max. in the synthetic sample (Avg. from experimental data)

The open-source Voronoi tessellations software, Neper v4.0.2 (Quey et al. 2011), was used to generate these synthetic samples using the “grain-growth” feature. Voronoi tessellations have been widely used to model polycrystal microstructures of rocks in 2D and 3D (Abdelaziz et al. 2018; Zhou et al. 2020). After constructing the synthetic samples, a script written in Python 3.0 environment (Van Rossum and Drake 2009) was used to assign mineral phases to the grains using a pseudo-random method, while also accounting for the relative sizes of different minerals in the samples. We constructed 61 × 137.25 mm^2^ synthetic grey granite, granodiorite, and pegmatite samples containing 3135, 18,948, and 36 grains, respectively. The size of the synthetic samples resembles the samples tested by Eberhardt et al. (1999). For the grain size analysis, we constructed synthetic samples with grain sizes ranging from 0.5 mm to 10 mm. The synthetic samples were then meshed using a Delaunay mesh geometry in Gmsh v4.4.1 (Geuzaine and Remacle 2009) having an average nominal mesh size of 0.3 mm for samples with average grain size ≤ 1.7 mm and 0.5 mm mesh for others. Mesh coarsening resulted in shorter runtime without compromising the accuracy of results (Abdelaziz et al. 2018). Acoustic emissions were monitored in the synthetic samples during numerical simulation utilizing the acoustic emission modeling algorithm in Lisjak et al. (2013). The algorithm simulates acoustic emissions in FDEM using a quasi-dynamic approach that is based on the relative displacements and kinetic energy of the cohesive crack elements in the proximity of the propagating cracks. The breakage of each crack element is assumed to be an acoustic event with its location coincident with the centroid of the element itself. The fracture mode is derived from the relative displacement of the fracture edges. The synthetic samples were simulated using Irazu FDEM software by Geomechanica Inc. (Lisjak et al. 2018).

Uniaxial load was applied with two platens moving towards each other at a velocity of 0.125 m/s, producing an equivalent strain rate of 1.8 s^−1^. This velocity is high compared to laboratory testing, however, the analysis showed that it provides reasonable run time without compromising the results (Mahabadi et al. 2014). The friction coefficient between the sample and platens was set to 0.1 to reduce the effect of interface friction on the macroscopic behavior. The models were run under plane-strain conditions, and a time step size of the order 10^–8^–10^–7^ s was used to ensure the stability of the explicit time integration scheme.

### Model Calibration

In the GB-FDEM framework, micromechanical parameters can be assigned separately to the individual grains and the grain boundaries, including the homophase (grains made of the same mineral) and the heterophase (grains made of different minerals) grain boundaries. This allows modeling the heterogeneity of granitic rocks imposed by different minerals and the contrast along their boundaries while capturing microfracturing mechanisms during the loading process. Physical properties of mineral grains such as density, elastic modulus, cohesion, tensile strength, and friction coefficient were obtained from experimental literature to realistically model the emergent behavior. Since the grain interface properties are experimentally challenging to obtain, grain boundary interfaces were calibrated through a trial-and-error process to obtain the macroscopic mechanical behavior. However, the solution is non-unique and calls for an extensive calibration process to obtain the numerical micromechanical parameters capable of simulating the emergent micromechanical response and macroscopic behavior. In addition, to better constrain the numerical parameters, we also considered matching the damage stress thresholds and fracture pattern for three granitic rocks from the same underground research laboratory but with different grain size distributions. With this approach, we ensure the robustness of the proposed model behavior in response to the varying grain size and mineralogical composition.

For this work, we adopted the same micromechanical parameters for each mineral, and the micromechanical parameters were calibrated using grey granite. After calibrating the grey granite synthetic sample, the same values were adopted for pegmatite and granodiorite. Thus, restricting our analysis to the effect of grain size and mineralogy percentages on the macroscopic mechanical response. These properly calibrated micromechanical properties of the synthetic samples resulted in emergent mechanical properties, discussed later in Sect. 3, well within literature-documented ranges (Abdelaziz et al. 2018; Mahabadi et al. 2012; Mavko et al. 2009). Mechanical parameters including CI, CD, UCS, and E values obtained from the experimental data are reported in Table 2.

**Table 2:** Mineral phases and grain boundaries properties for synthetic grey granite, granodiorite, and pegmatite (Fsp—feldspar, Bt—biotite, Qz—quartz)

Property (unit)	Fsp	Bt	Qz
Density (kg/m^3^)^1^	2600	2800	2600
Young’s modulus (GPa)^2^	60	29.3	83.1
Poisson ratio (−)^2^	0.32	0.36	0.17
Friction coefficient (−)^3^	1.27	1.27	1.27
Cohesion (MPa)^3^	33	30	35
Tensile strength (MPa)^3^	8	5.5	14
Mode I fracture energy (J/m^2^)^4^	390	599	907
Mode II fracture energy (J/m^2^)^4^	690	1198	1810
Fracture penalty (GPa)^4^	564	172	832
Normal penalty (GPa/m)	112	344	166
Tangential penalty (GPa/m)	1120	344	1660

Grain boundary	Homophase			Heterophase		
Property (unit)	Bt-Bt	Fsp-Fsp	Qz-Qz	Bt-Fsp	Bt-Qz	Qz-Fsp
Friction coefficient (−)	1.14	1.14	1.14	0.81	0.81	0.81
Cohesion (MPa)	27	31	31.5	32.5	31	32
Tensile strength (MPa)	4.95	7.2	12.6	8.5	8.5	2.0^*^
Mode I fracture energy (J/m^2^)	449.25	1.98	680.25	1.81	1.07	300
Mode II fracture energy (J/m^2^)	898.5	465.0	1360.5	382	382	1450
Fracture penalty (GPa)	3440	5640	8320	860	86	800
Normal penalty (GPa.m)	6880	1120	1660	172	172	600
Tangential penalty^**^ (GPa/m)	6880	1120	1660	172	172	600

^1^Mavko et al. (2009), ^2^Villeneuve (2008), ^3^Mahabadi (2012), ^4^Abdelaziz et al. (2018)

*Lower tensile strength than Bt-shared boundaries was assumed to mimic the higher density of pre-existing cracks along Qz-Fsp boundaries in thin sections (e.g., Ghasemi et al. (2020))

**Equally high tangential and normal penalty values were used to ensure stiff behavior of grain boundaries

### Damage Stress Thresholds Analysis

Damage stress thresholds are crucial for rock damage assessment and failure prediction. These thresholds are commonly inferred using the strain-based method (SBM) and acoustic emission method (AEM) (Diederichs et al. 2004; Eberhardt et al. 1999; Zhang et al. 2020). In this paper, both SBM and AEM were used to infer and assess the damage stress thresholds.

In the SBM, the total volumetric strain (ε_Vtot_) of a uniaxially loaded sample is composed of the elastic volumetric strain (ε_Vel_) and the inelastic volumetric strain, which is commonly called the crack-volumetric strain (ε_Vcr_) (Martin and Chandler 1994). Crack volumetric strain is calculated as:

$${\varepsilon }_{V{\text{cr}}}= {\varepsilon }_{V{\text{tot}}}-{\varepsilon }_{V{\text{el}}}$$

$${\varepsilon }_{V{\text{el}}}= \frac{1-2v}{E}{\sigma }_{1}$$

where $\nu$ and *σ*_*1*_ are the Poisson’s ratio and applied stress, respectively. It is noteworthy that the volumetric strain is represented by areal strain under the plane-strain 2D simulations. In this numerical analysis, volumetric strains are calculated using two methods. In the first method, the nodes forming the boundary of the synthetic rock samples are traced at each time step to calculate total volumetric change (assuming unit thickness) using the convex hull method (Barber et al. 1996). In the second method, a virtual strain gauge (SG) of 1 cm length spans from the center of the synthetic sample horizontally and vertically to measure the axial ${\varepsilon }_{{\text{ax}}}$ and lateral ${\varepsilon }_{{\text{lat}}}$ strains, respectively, and calculate the volumetric strain using ${\varepsilon }_{V{\text{tot}}}\approx {\varepsilon }_{{\text{ax}}}+{\varepsilon }_{{\text{lat}}}$ (ASTM 2013) (only ${\varepsilon }_{{\text{lat}}}$ was added instead of ${2\varepsilon }_{{\text{lat}}}$ due to the 2D simulation). Crack initiation using SBM (CI-SBM) was inferred from the initial change in the slope of the crack-volumetric and lateral strains while the crack damage (CD-SBM) threshold was taken at the maximum total volumetric strain (Bieniawski 1967; Brace et al. 1966; Martin 1993). It is worth mentioning that the values of CI-SBM and CD-SBM are highly biased by the calculation of the elastic parameters especially Poisson’s ratio and mesh elements that may have dislodged from the model.

In the AEM, damage stress thresholds (i.e., CI-AEM and CD-AEM) were determined based on the change of cumulative AE count with the applied stress. Using this method, the initial increase in cumulative AE count, which appears as the first deviation from the horizontality of the cumulative AE curve, is taken as the CI-AEM threshold. Since the synthetic sample being modeled is intact, the small number of AEs that typically occur at the crack closure stage in laboratory experiments were not considered. Furthermore, the deviation from the linearity before the peak strength marks the CD-AEM threshold which defines the onset of unstable crack propagation associated with a large increase in AE count.

### Effects of Grain Size on Mechanical Behavior

The evolution of mechanical properties with different mean grain sizes was studied through a parametric study of 18 synthetic granite samples. A small standard deviation was used in generating these synthetic samples, thus avoiding uniform or absurdly large variations in grain size and ultimately avoiding the effects of grain size heterogeneity (Liu et al. 2018; Peng et al. 2017). Typical mineralogical compositions of 65% feldspar, 10% biotite, and 25% quartz were used throughout the samples using the mineral properties stipulated in Table 2. The loading rate used in the calibration stage was used for this study, and all simulations were carried out in unconfined conditions.

### Effects of Mineralogical Composition on Mechanical Behavior

Effects of mineralogical composition on the mechanical properties of granite were systemically analyzed using 13 synthetic samples with random mineral compositions as shown in Table 3, in which quartz content varied from 5 to 43.5%, biotite content varied from 2 to 15%, and feldspar content varied from 52 to 89%. These ranges of values generally resemble the various possible mineral compositions of granite rocks found in nature (He et al. 2023; Sajid et al. 2016; Tuğrul and Zarif 1999). All simulations were carried out in unconfined conditions using loading rates as those used in the calibration stage.

**Table 3:** Composition of the synthetic granite samples with random mineral assignment used in the study

Comp. (%)	M1	M2	M3	M4	M5	M6	M7	M8	M9	M10	M11	M12	M13
Quartz	31	29	36	20	25	30	15	10	5	43.5	5	30	37
Biotite	10	15	4	5.5	5	7	5	5	6	4.5	10	2	6
Feldspar	59	56	60	74.5	70	63	80	85	89	52	85	68	57

## Results

### Experimental Results

**Fig. 3:**
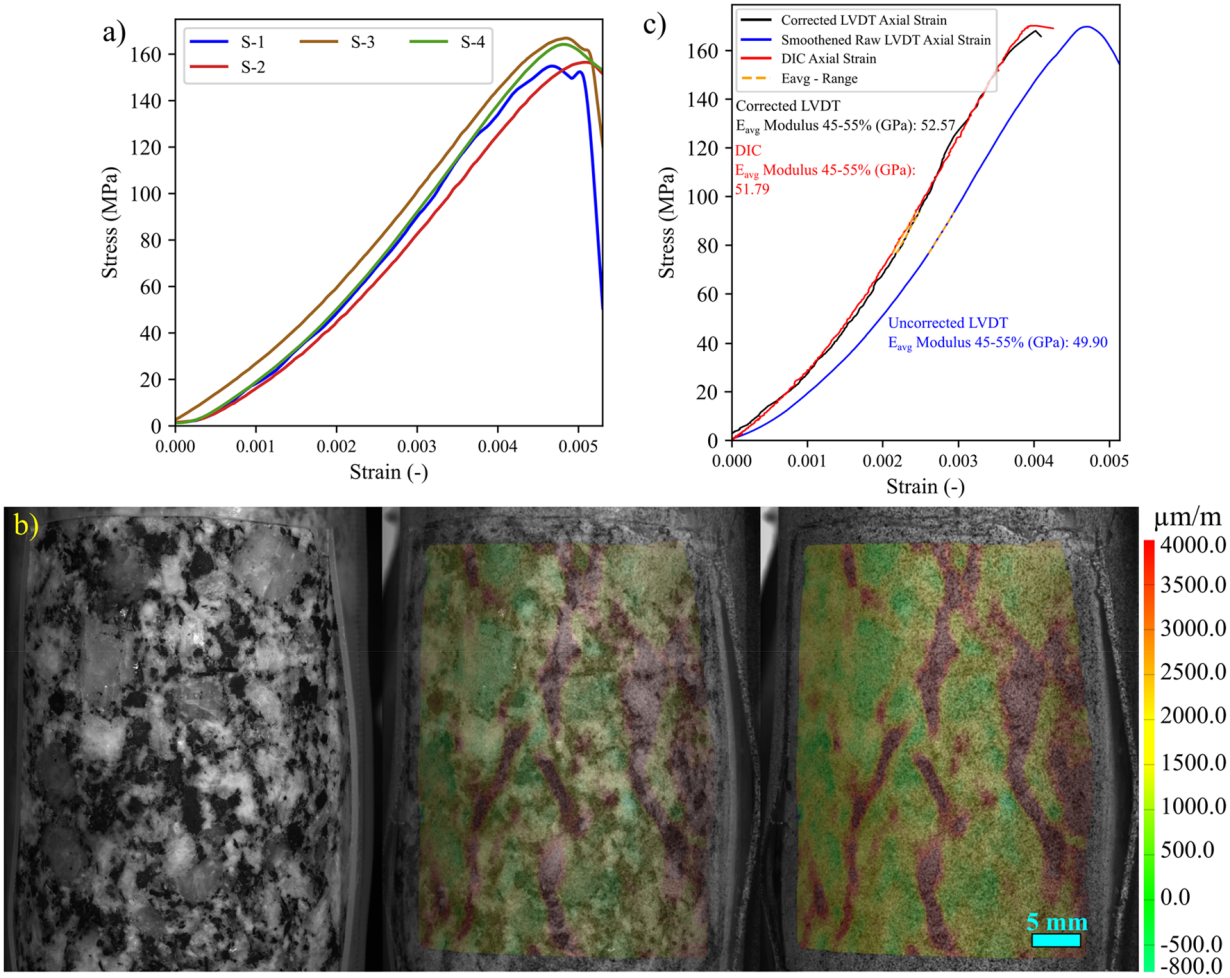
**a** Stress–strain diagrams, using the strain captured by the virtual DIC LVDTs, for all the tested samples. **b** Major principal strain field at 90% UCS overlaid on the S-1 sample image. Major strain field and macrocracks development correlate with the heterophase grain interfaces. **c** Complete stress–strain curve for sample S-4. Strain is represented using the data captured by the LVDT, the LVDT data after being corrected for system compliance (Abdelaziz and Grasselli 2021), and the data extracted using the DIC virtual LVDT

Complete stress–strain diagrams, using the strain captured by the virtual DIC LVDTs, for the four tested samples are shown in Fig. 3a. Major strain localization indicates that macrocracks preferentially propagated along different minerals grain boundaries; mainly the shared boundaries with biotite grains (Fig. 3b). Stiffness contrast between the compliant biotite grains and the adjacent stiff feldspar and quartz grains provided nuclei for crack propagation. This strain localization pattern also indicates higher intergranular cracking occurrence during the uniaxial loading of granitic rocks where few intragranular strain localization was observed in the larger feldspar grain (Fig. 3b). Figure 3c sheds context to the validation of the strain data for the tested sample obtained using the strain measured by the physical LVDTs and those captured by the virtual DIC LVDTs. The strains captured by the physical LVDTs showed an elastic modulus of 49.90 GPa, while the elastic modulus calculated using the DIC data was 51.79 GPa. Although the elastic modulus is similar, the magnitude of the strain values obtained by the LVDT are about 25% more (Fig. 3c). However, when the DIC is compared to the LVDT after being corrected for system compliance (i.e., the strain of the test setup) (Abdelaziz and Grasselli 2021) the magnitude of the strain is comparable. In addition, the calculated E from the physical LVDTs and the virtual DIC LVDTs are 52.57 and 51.79 GPa, respectively (Fig. 3c).

**Fig. 4:**
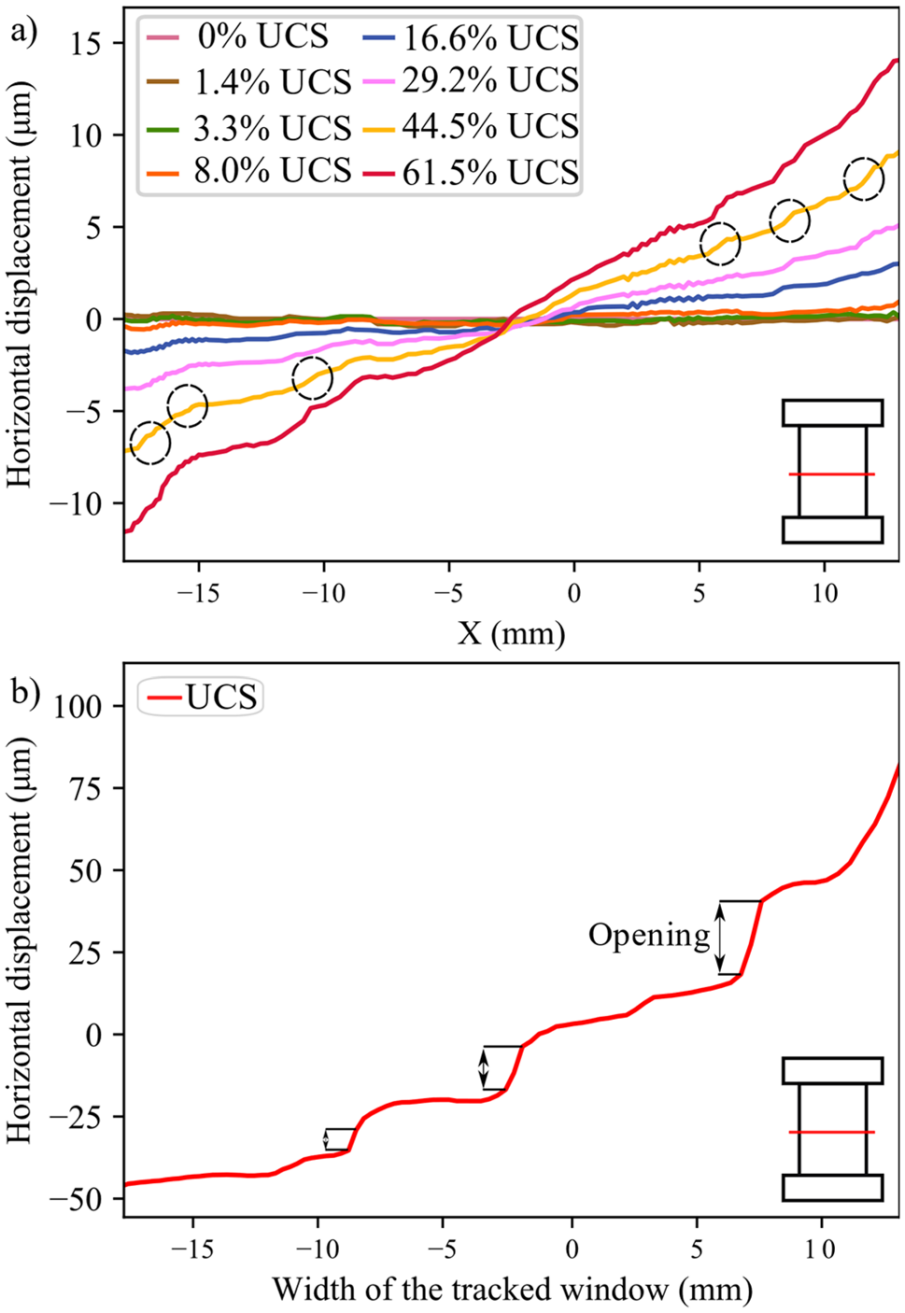
**a** Horizontal displacement along sample S-1 width taken at the middle of the tracked window. Dashed circles show cracks that appeared as jumps on the horizontal displacement curve. **b** Horizontal displacement at the middle of the S-1 sample at the peak strength. The difference in the horizontal displacement across displacement discontinuity was used to estimate crack opening

**Fig. 5:**
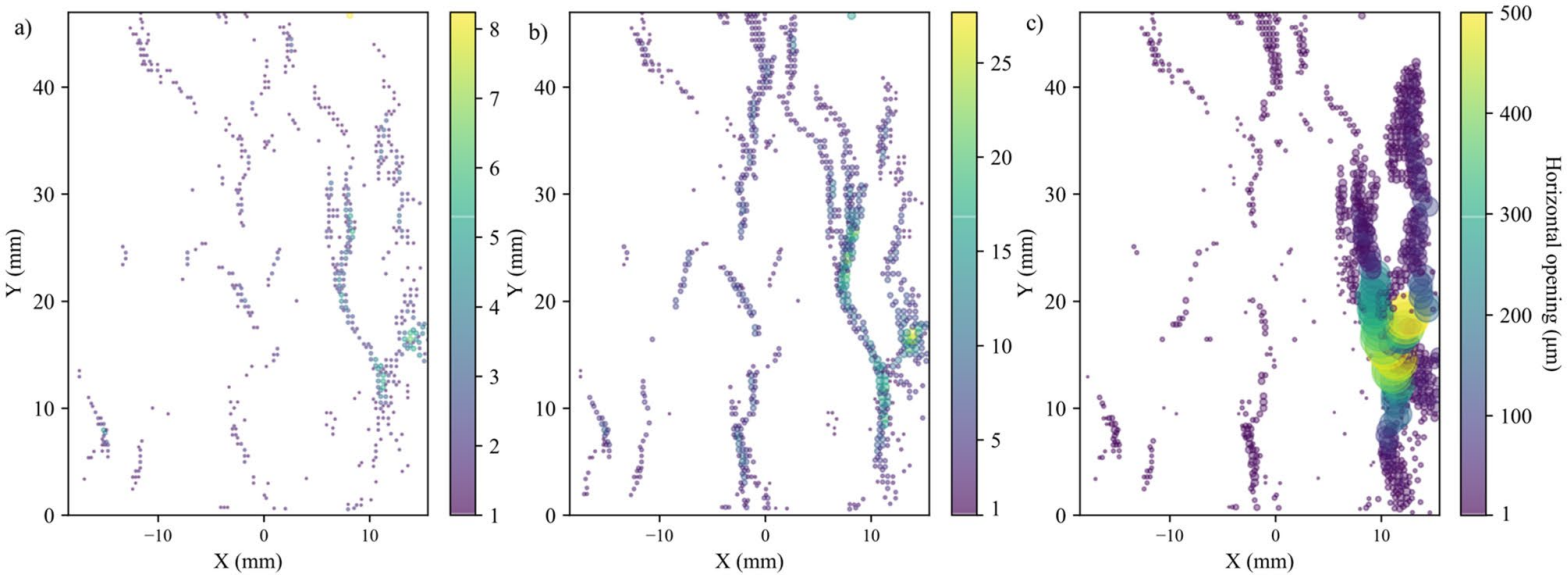
Horizontal opening in sample S-1 (indicated with color and relative symbol size) of macrocracks at **a** CD (84% UCS), **b** peak strength, and **c** 4% post-peak

**Fig. 6:**
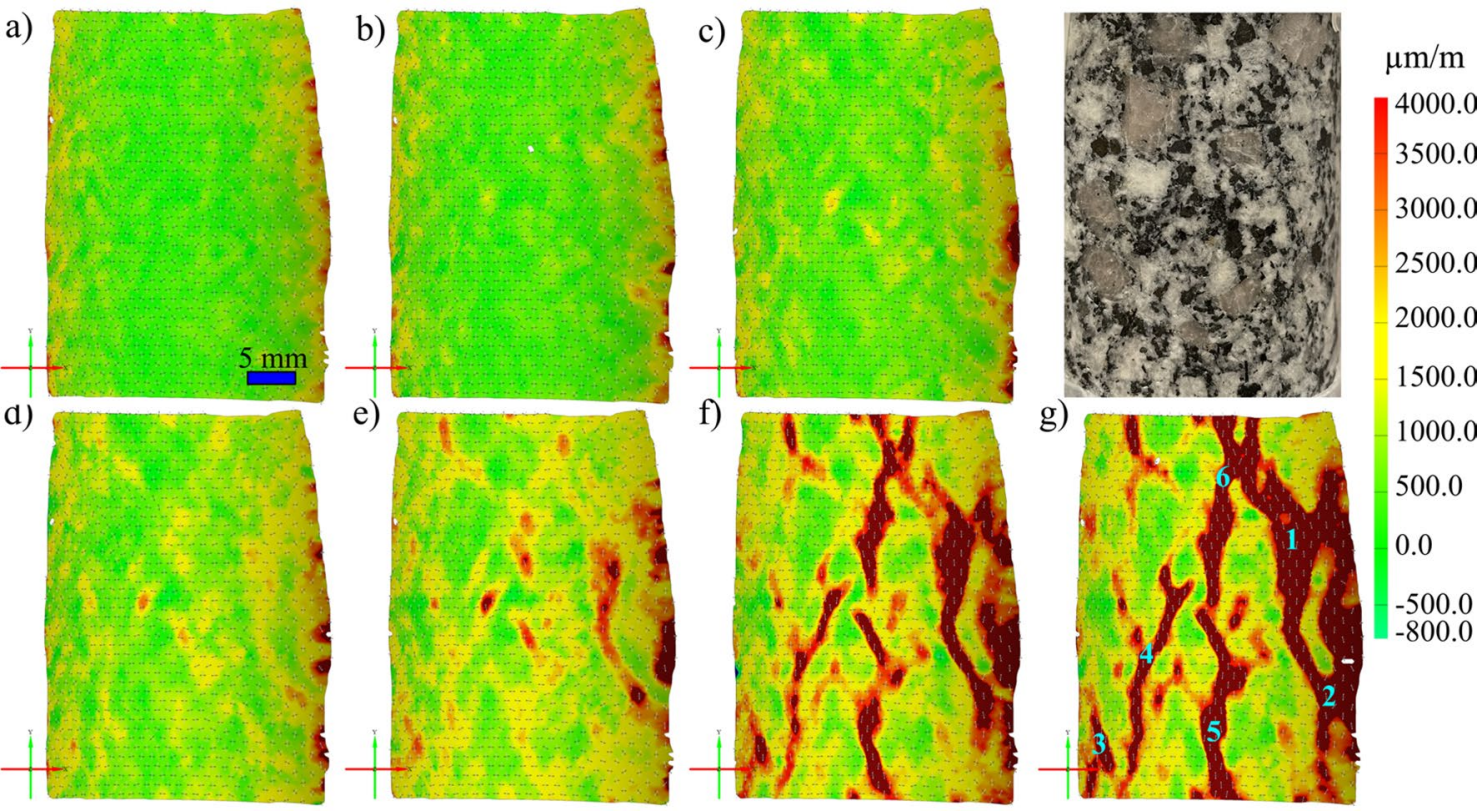
Major principal strain field in sample S-1 at **a** 44.5, **b** 60, **c** 70, **d** 77.5, **e** 84, **f** 95, and **g** 100% UCS. Numbers in **g** indicate the locations of the tracked crack openings on S-1

**Fig. 7:**
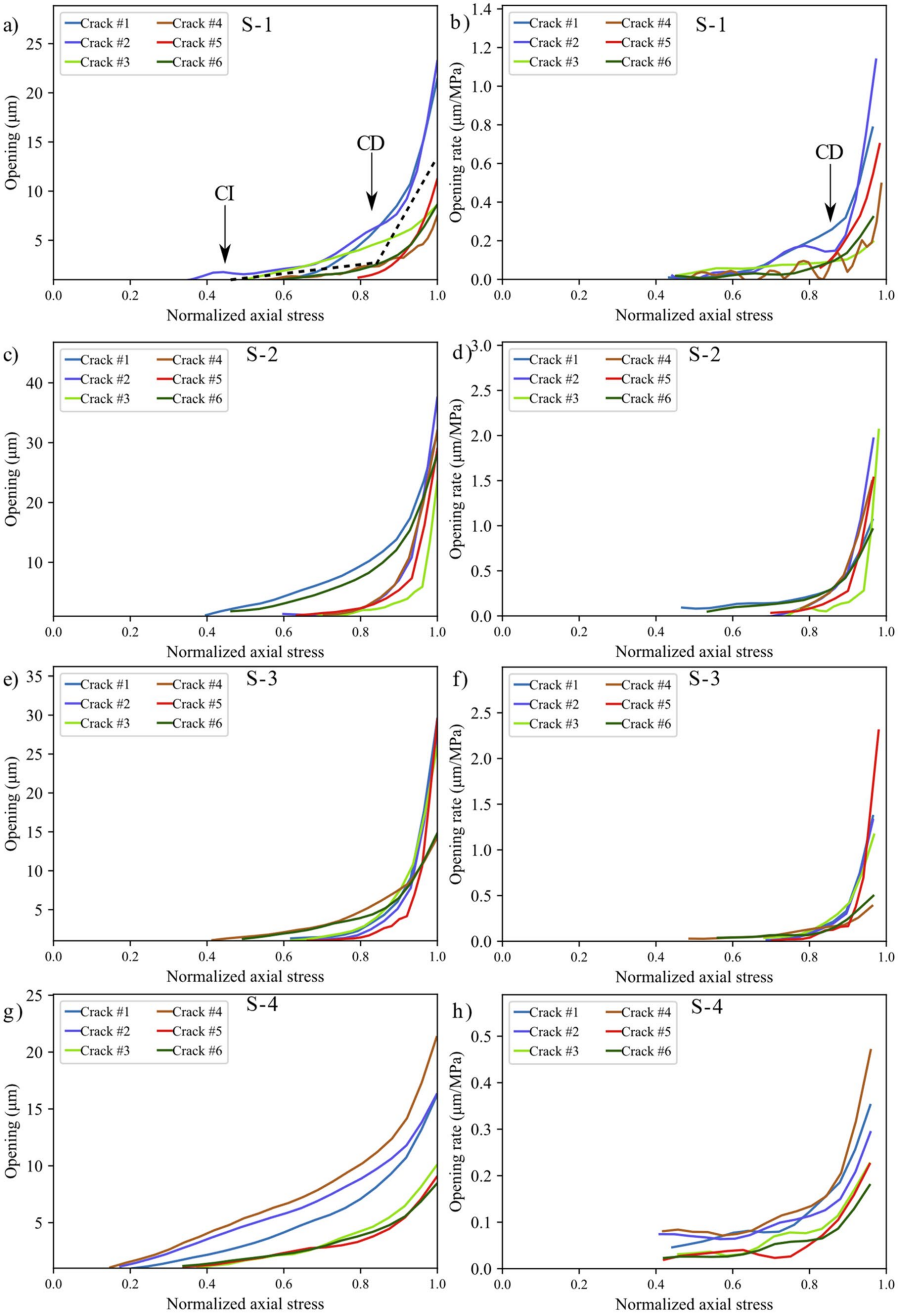
Evolution of crack opening and crack opening rate during the uniaxial loading of samples S-1 **a** and **b**, S-2 **c** and **d**, S-3 **e** and **f**, and S-4 **g** and **h**. Corresponding cracks in a and b are shown in Fig. 6 for sample S-1

**Fig. 8:**
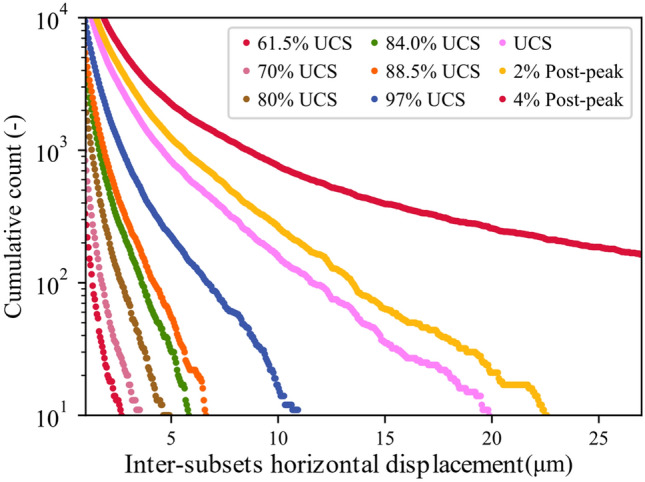
Evolution of horizontal displacement between tracked subsets at different stages around the UCS for sample S-1

**Fig. 9:**
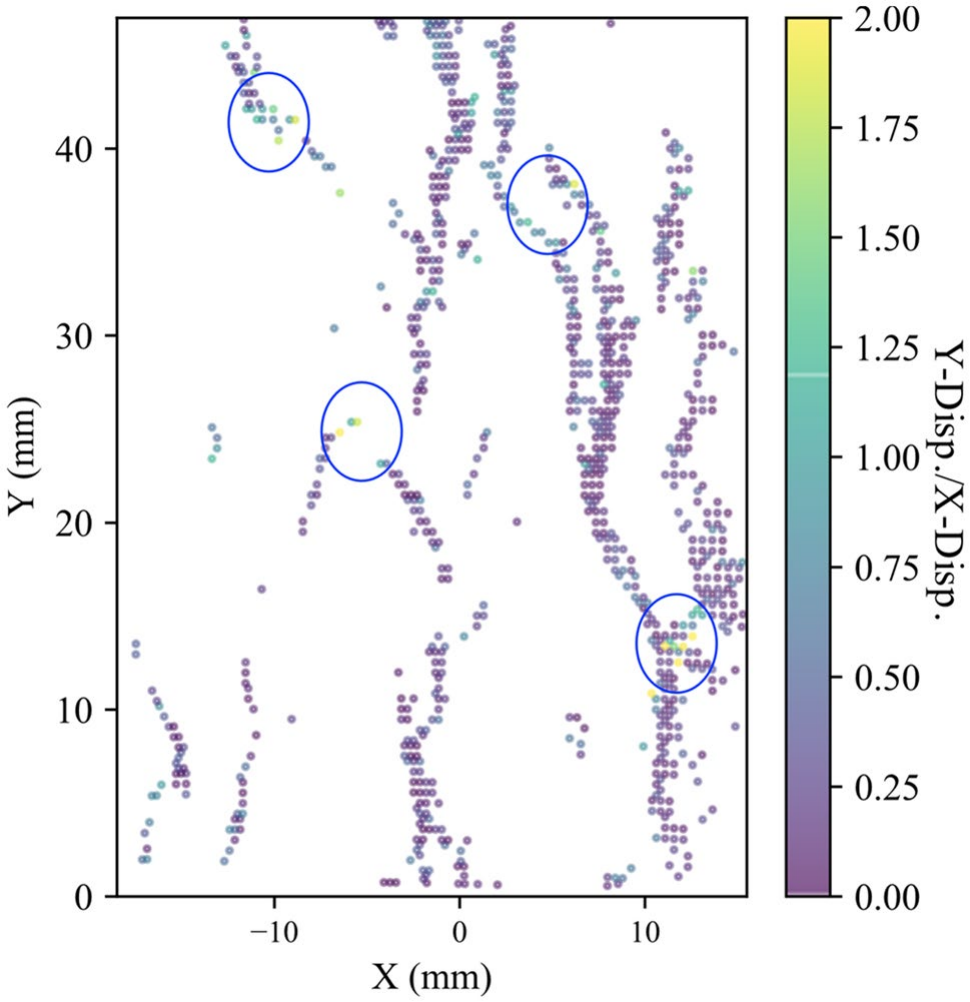
Ratio of the displacement in Y and X directions of the cracks at peak strength for sample S-1. Blue circles show potentially shear-dominated cracks

The stress–strain curves for all the tested samples, depicted in Fig. 3, indicate that the behavior of the samples was similar. In addition, all the samples exhibited similar strain localization patterns and consistent failure patterns (Fig. S1 to Fig. S3 in Supplementary material). Hence, sample S1 will be used as the representative sample and its results discussed in-depth. For sample S-1, with the commencement of the uniaxial compression, horizontal displacement starts to increase due to Poisson’s effect as can be seen on the horizontal displacement curves at 1.4—29.2% UCS (Fig. 4a). As the stress level increases, cracking events can be observed on the horizontal displacement curves as jumps (or discontinuities). The relatively larger jumps on the horizontal displacement curve at 44.5% UCS (69.0 MPa) during the loading convincingly suggest the crack initiation (CI) stage (Fig. 4a). Figure 4b illustrates how the crack opening was estimated from the horizontal displacement field. Openings of macrocracks at the crack damage (CD) threshold, peak strength, and post-peak are shown in Fig. 5. It can be noticed that each damage zone consists of several microcracks as reflected by the multiple openings within each zone (Fig. 5). This behavior is apparent in the width of the strain localization zone in Fig. 6. Opening of cracks increased with the applied load and accelerated at the crack damage threshold at around 84% UCS (131 MPa) (Fig. 7). The cumulative count of horizontal displacement between tracked subsets shifted towards higher values with the loading progression with a significant increase after the CD threshold (Fig. 8). Since it is challenging to identify shear and tensile cracks in heterogenous rocks such as granite using DIC with certainty, we used the Y-displacement-to-X-displacement ratio (Fig. 9) as a proxy to the potential failure mode. The higher this displacement ratio (i.e., more sliding than opening) the higher the probability of shear failure, while the lower the ratio (i.e., more opening than sliding), the higher the probability of tensile failure is. The displacement ratio of the cracks at the peak strength indicates a tensile-dominated cracking pattern while few shear-dominated cracks can be observed at high angles to the applied axial load (Fig. 9). CI for samples S-2, S-3, and S-4 is 40.0, 39.0, and 30.0% UCS, respectively, while the CD is 83.0, 80.8, and 85.0% UCS for those samples, respectively.

### Calibrated Synthetic Samples

**Fig. 10:**
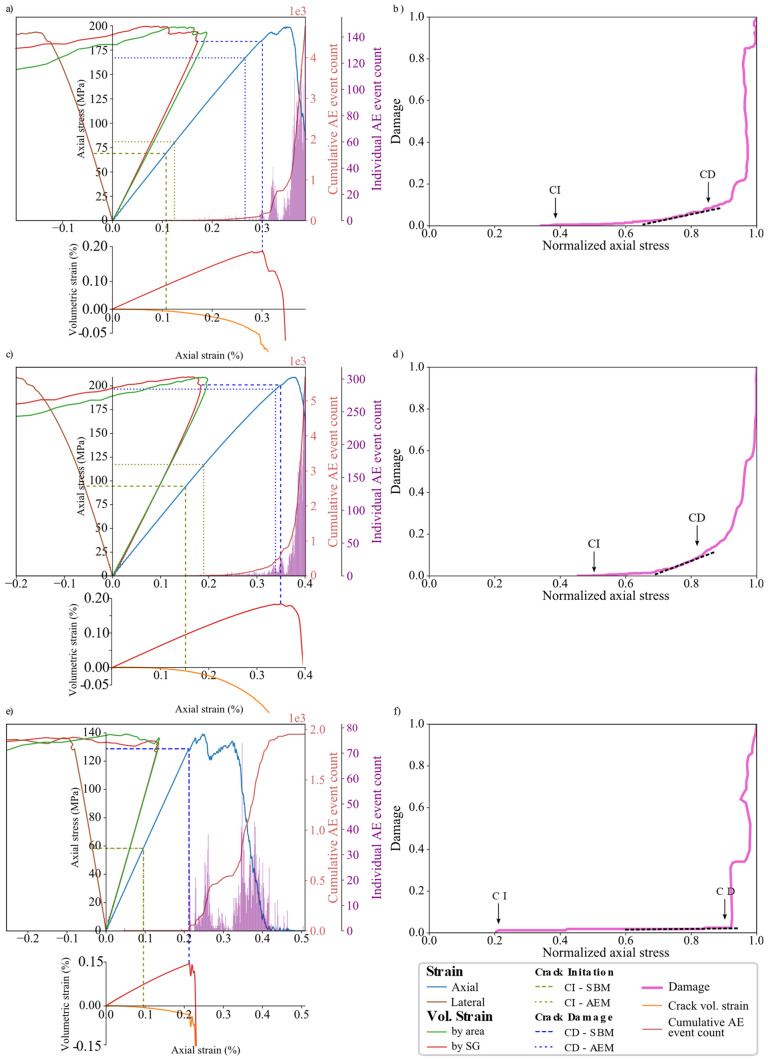
Complete stress–strain diagrams, AE data, and damage evolution for **a** and **b** synthetic grey granite, **c** and **d** granodiorite, and **e** and **f** pegmatite. Total volumetric strain is calculated from the variation in boundary node tracing, i.e., model area, (green solid line) and from virtual strain gauges to measure lateral and axial strains (red solid line). Crack initiation (CI) and crack damage (CD) stress thresholds were inferred from both strain data and acoustic emissions. CD-AEM thresholds were picked from the deviation from the linear trend of the damage curve (black dotted line). (For interpretation of the references to color in this figure legend, the reader is referred to the web version of this article.)

The results of the UCS and E for the three synthetic samples fall within 0.5% and 8.4%, respectively, of their experimental values (Table 4). Pegmatite is characterized by large grain size (up to a few centimeters) which results in varying peak strength values depending on the minerals grain spatial distribution and orientation with respect to the applied load in the tested synthetic sample (discussed later in Sect. 4). Figure 10 shows the complete stress–strain curves for the synthetic samples and the associated AE events. The level of damage, indicated by the ratio of cumulative AE to the cumulative AE at peak strength, is plotted versus axial stress normalized to the sample’s UCS (Fig. 10). Damage stress threshold picks using SBM and AEM are overlaid on the complete stress–strain curves illustrated in Fig. 10. Values of the CI and CD thresholds for the synthetic samples and their experimental counterparts are reported in Table 5. CD-AEM values show a good match with the experimental values. However, CD-SBM values inferred from virtual strain gauges are lower compared to those from AEM. In Fig. 10, damage stress thresholds using AEM are illustrated on the cumulative AE with the normalized axial stress plots for the three synthetic samples.

**Table 4:** Comparison between the numerical and experimental UCS and E

	UCS (MPa)			E (GPa)		
	Num	Exp	Diff. (%)	Num	Exp	Diff. (%)
Grey granite	198.8	200.0	0.5	64.1	63.8	0.5
Granodiorite	209.5	221.5	5.0	61.6	67.2	8.4
Pegmatite	139.15	145.1	4.3	61.2	60.1	1.8

**Table 5:** Comparison between the numerical and experimental CI and CD thresholds

	CI (MPa)			CD (MPa)		
	SBM	AEM	Exp	SBM	AEM	Exp
Grey granite	71	76.7	79.6	162.7	167.8	147.4
Granodiorite	95	115	110	141.3	193.5	194.0
Pegmatite	56	56	72.0	128.8	128.8	113.2

**Fig. 11:**
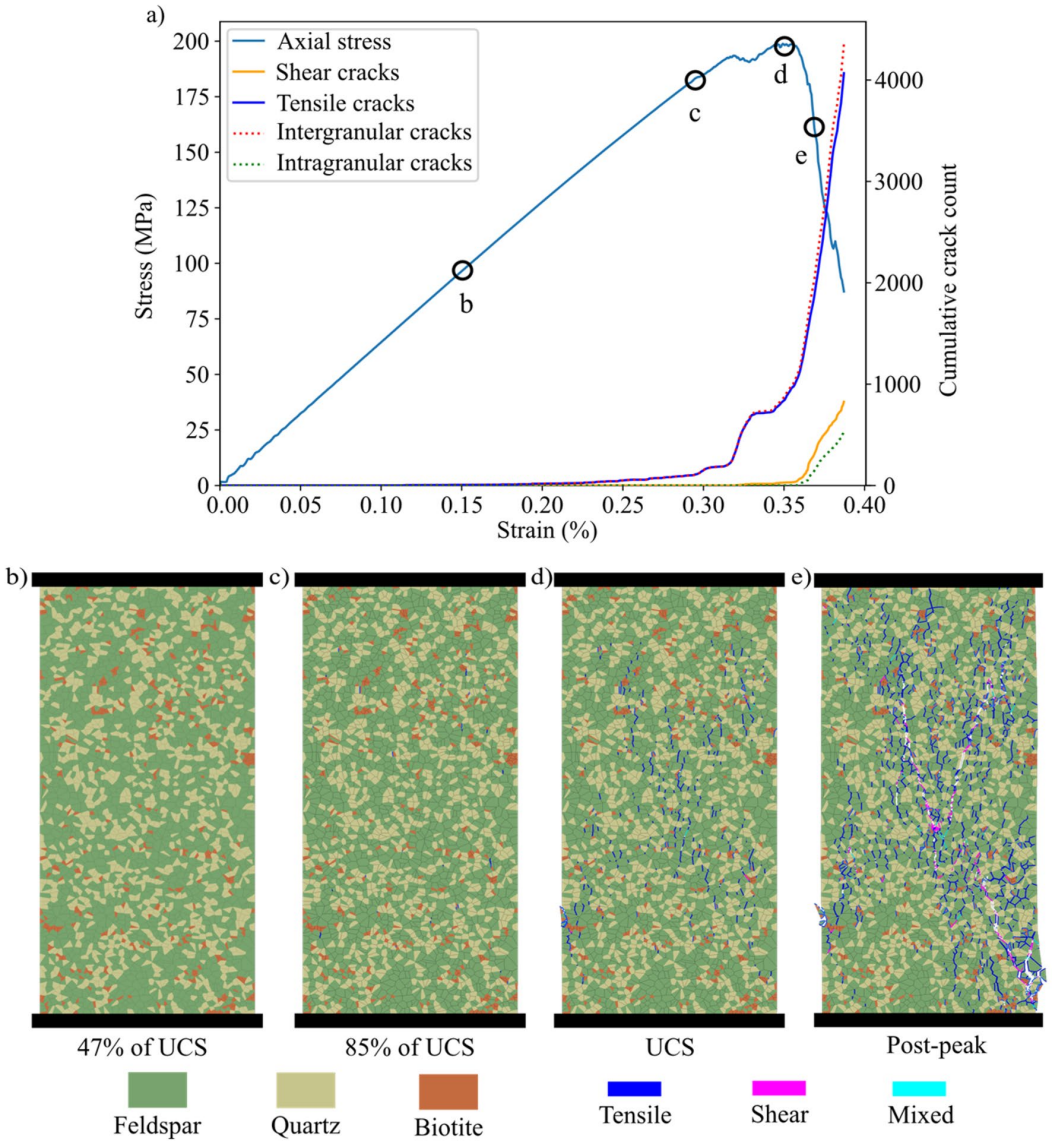
**a** Evolution of intragranular, intragranular, tensile, and shear cracks count with the applied axial stress for grey granite synthetic sample. Macroscopic fracture evolution with the applied axial stress in grey granite synthetic sample at **b** 47% UCS, **c** 85% UCS, **d** UCS, and **e** post-peak (162 MPa)

**Fig. 12:**
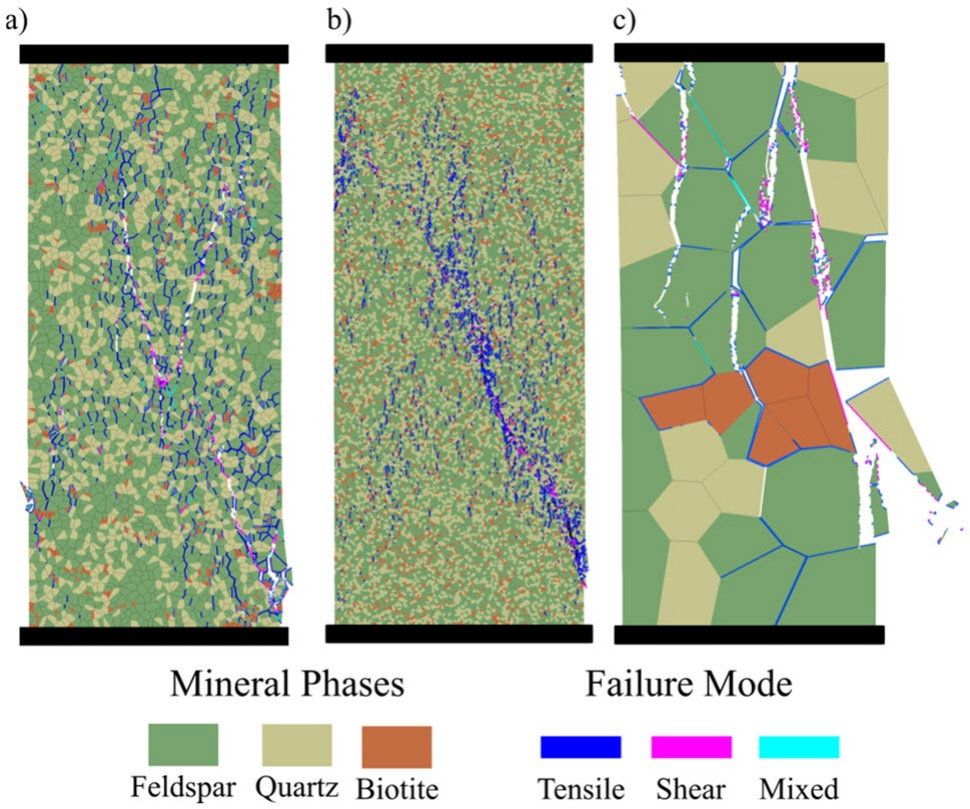
Macroscopic and microscopic failure modes of **a** synthetic grey granite, **b** granodiorite, and **c** pegmatite. Grey granite and granodiorite show shear bands while pegmatite shows axial splitting

Figure 11a shows the evolution trends of crack type and mode in the grey granite synthetic sample during the compressive loading. Figure 11b-e illustrates the macroscopic fracture evolution during the complete stress–strain of the grey granite synthetic sample. The macroscopic failure in grey granite and granodiorite occurred due to the formation of a clear shear zone while pegmatite underwent axial splitting, as depicted in Fig. 12.

### Microcracking Patterns

**Fig. 13:**
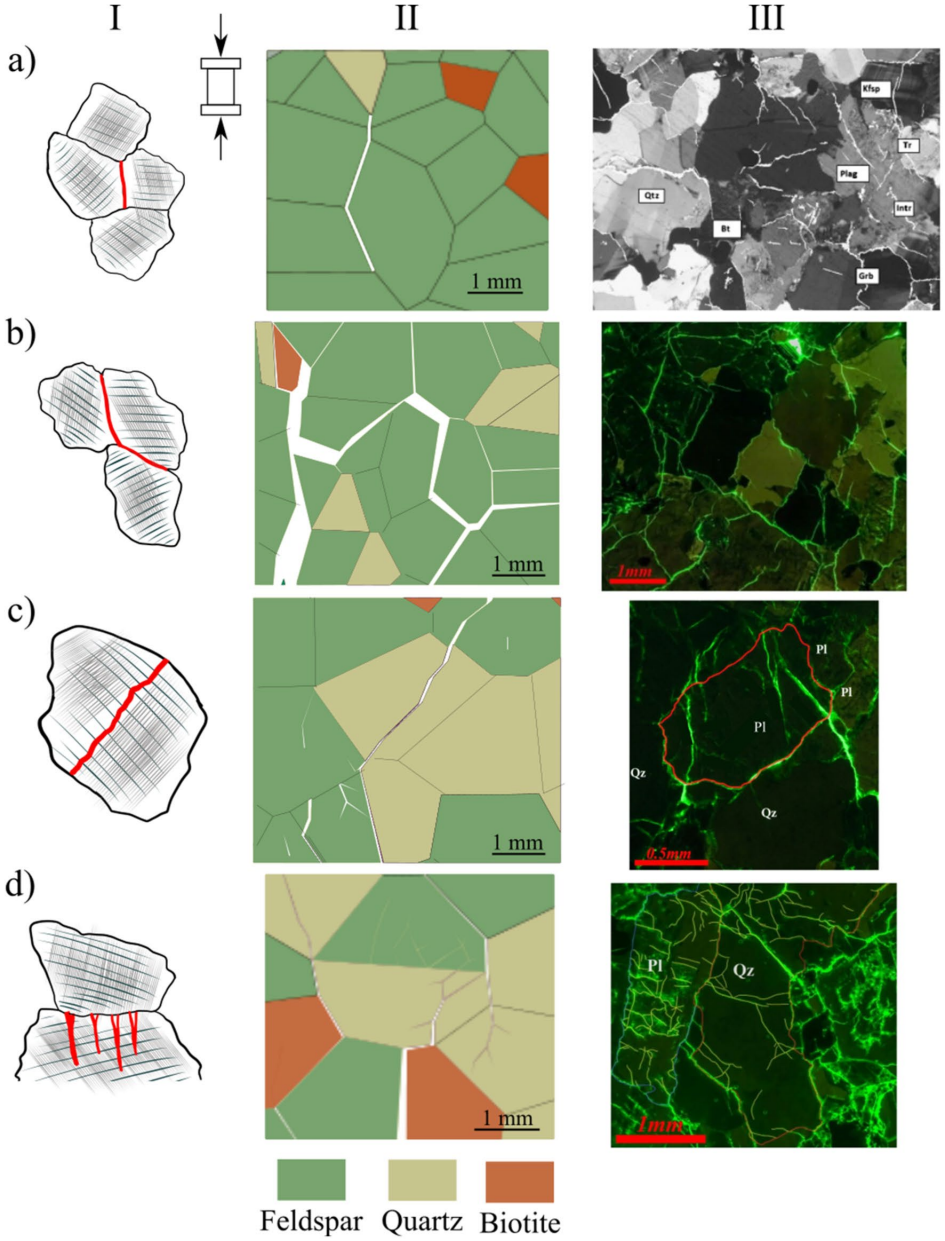
Microcracking patterns in brittle rocks: **a** intergranular tensile crack subparallel to the applied load, **b** intergranular sliding shear crack at a high angle to the applied load associated with tensile at the leading end, **c** intragranular shear crack, and **d** formation of high aspect-ratio fragments from tensile cracking due to stiffness-contrasting grains (column I denotes a conceptual grain-scale deformation model, column II represents GB-FDEM results, and column III is microscopic observations of the deformation process—Qz and Pl denotes quartz and plagioclase feldspar, respectively) ( III-a was modified from Lim et al. (2012) and III-b, -c, and -d were modified from Ghasemi et al. (2020))

The microcracking patterns observed in the simulations were almost identical. At early loading stages, intergranular cracks initiate typically subparallel to the loading direction due to extensional tensile stresses along grain boundaries. These early cracks usually result from stress concentration from angular grains indentation contacts causing a wedge effect (Fig. 13a), or from the tailing and leading edges of sliding grains (Fig. 13b). In the grey granite synthetic sample, cracks initiate at about 40% of the UCS. Intergranular cracks started first along boundaries between the stiff quartz and the more compliant biotite grains.

**Fig. 14:**
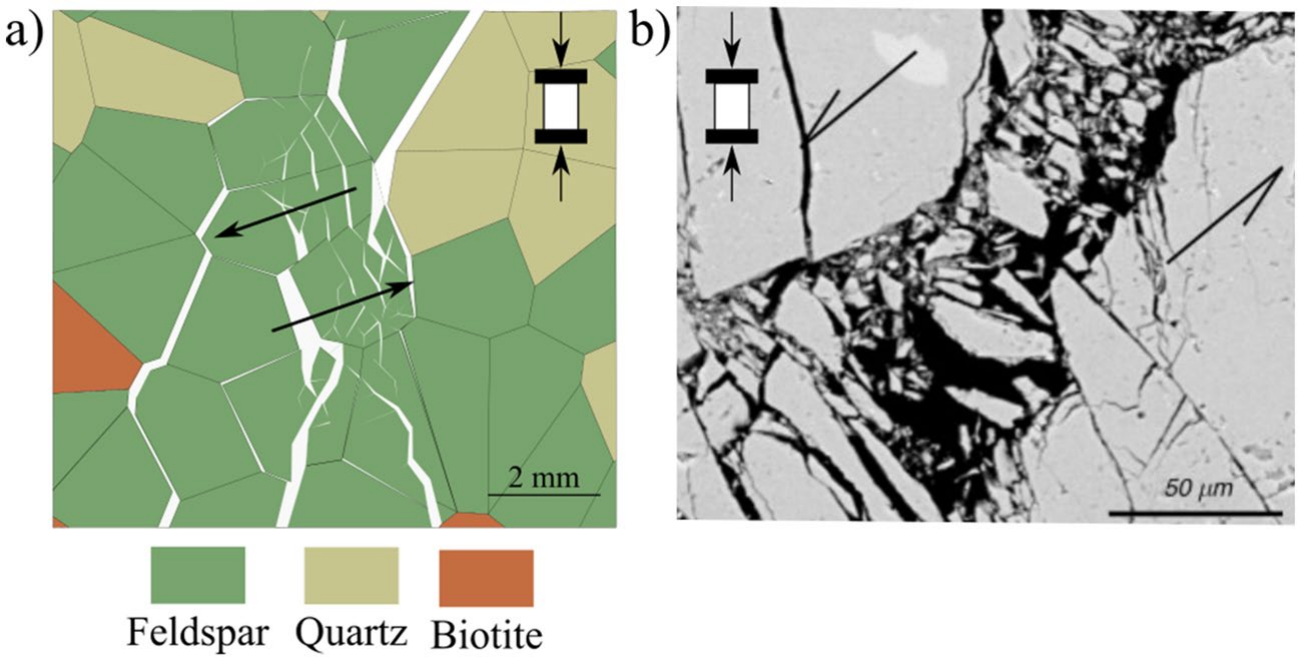
Fragmentation and rotation of feldspar grains in **a** the synthetic grey granite and **b** deformed granite sample visualized under the scanning electron microscope (SEM) (modified from Dresen and Guéguen (2004)). Loading in both images is in the vertical direction and arrows indicate shear zone orientation

At higher stress levels, more intergranular cracks initiate at larger angles to the applied load. With increasing stress levels, intragranular cracks appear in the form of extensional and sliding cracks inside the grains (Fig. 13c and d) and as the stress continues to increase these cracks propagate to adjacent grains to form transgranular cracks (Fig. 13c). These cracks form as a result of stress concentration from angular grain tips (Fig. 13a) or grain stiffness mismatch (Fig. 13d). Large grains break into columnar fragments parallel to the applied load (Fig. 13d). Furthermore, in the post-peak stage, stiff grains such as quartz and feldspar splinter to form the gouge zone along the formed shear zone (Fig. 14).

The synthetic samples showed an increase in the number of intragranular cracks with increased stress levels (Fig. 11a), while transgranular cracks appeared more frequently towards the post-peak. Transgranular cracks formation and coalescence of initiated cracks promoted damage more than the nucleation of new cracks. This can be noticed as damage localization in specific zones prior to sample failure (Fig. 12). Moreover, it was also observed that the dominant cracking mode under uniaxial loading was tensile-dominated failure (mode I) with few shear-dominated cracks (mode II) appearing after the crack damage threshold towards post-peak (Fig. 11d-e).

### Effects of Grain Size on Mechanical Behavior

**Fig. 15:**
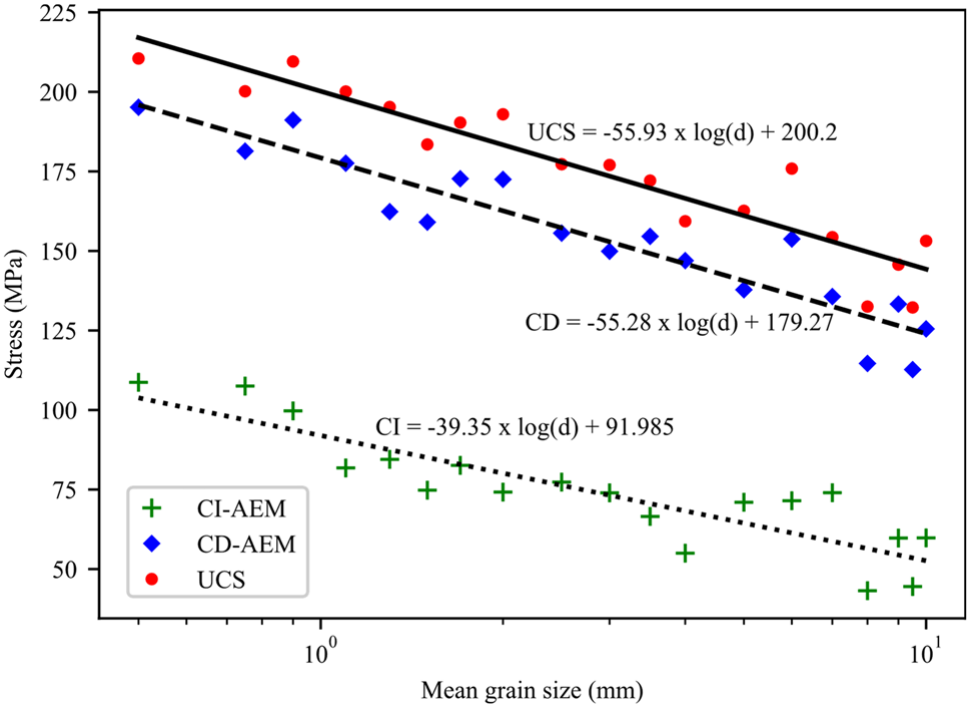
CI-AEM, CD-AEM, and UCS results for numerical samples with different mean grain sizes (d_grain_)

**Fig. 16:**
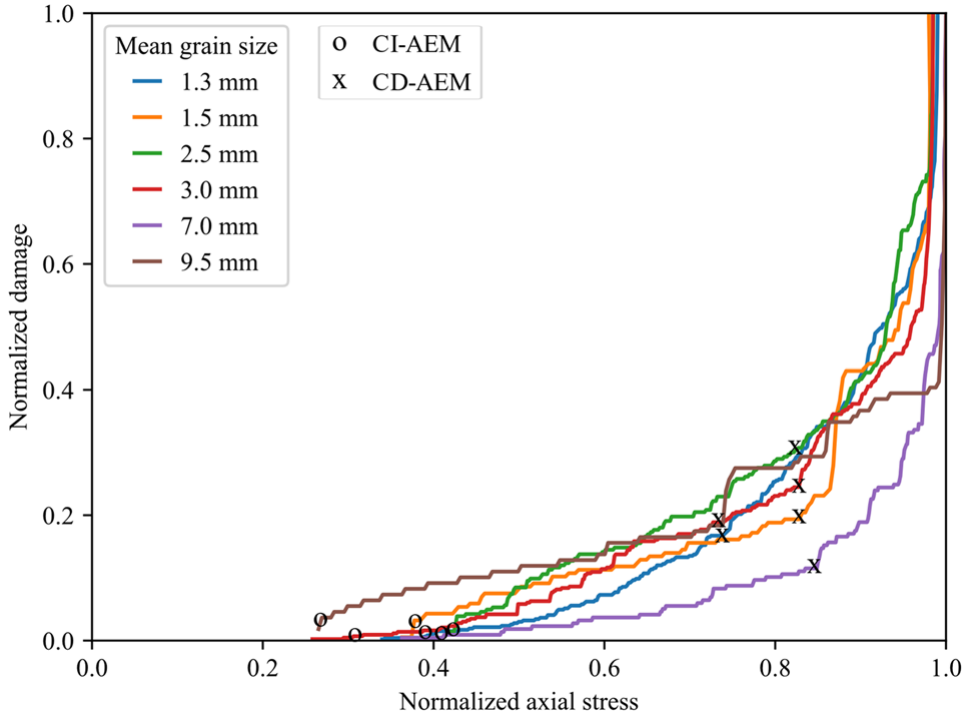
CI-AEM and CD-AEM results for numerical samples with different mean grain sizes (d_grain_)

**Fig. 17:**
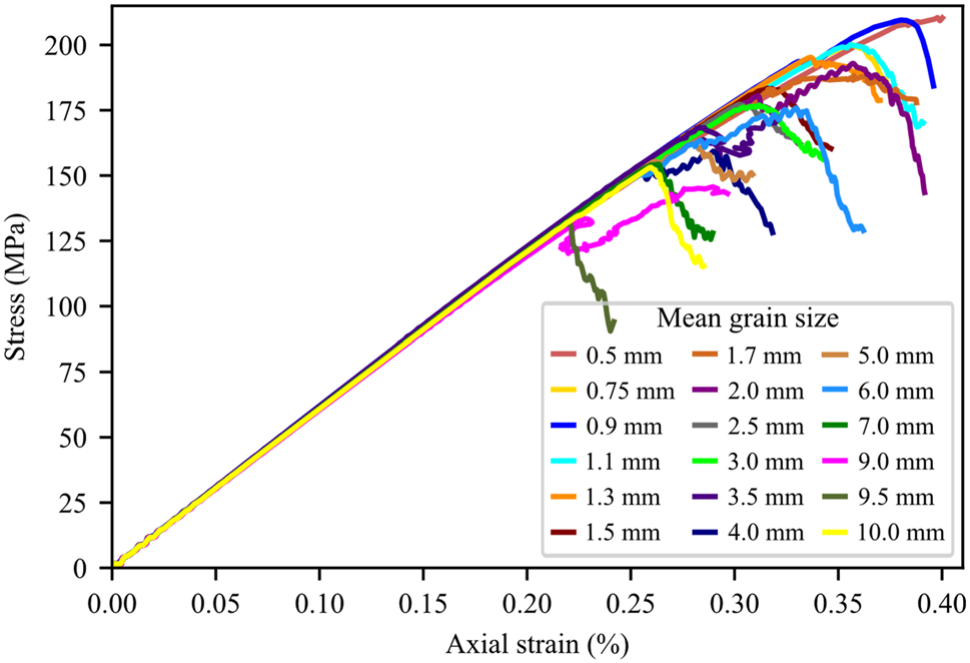
Complete stress–strain data for synthetic samples used for parametric grain size analysis

Results of the grain size effect show that the fine-grained synthetic granite samples exhibited higher strength than those with coarser grains (Fig. 15). Peak strength and crack stress thresholds data suggest a log-linear relation with the mean grain size (d_grain_) in the form UCS, CD, CI = A x log(d_grain_) + B which has been reported in the literature for peak strength. Slopes of the UCS, CD, and CI curves indicate that the dependencies on the mean grain size are similar for CD and UCS. Dependency, although lower than that of CD and UCS, is also seen for the CI. Figure 16 shows the analysis of AE data for 6 synthetic samples to infer CI and CD thresholds. We used the AEM method to infer these thresholds and a comparison between CD-AEM and CD-SBM is provided in Sect. 4. The complete stress–strain diagrams for the tested synthetic samples do not show any significant effect of grain size on E (Fig. 17).

**Fig. 18:**
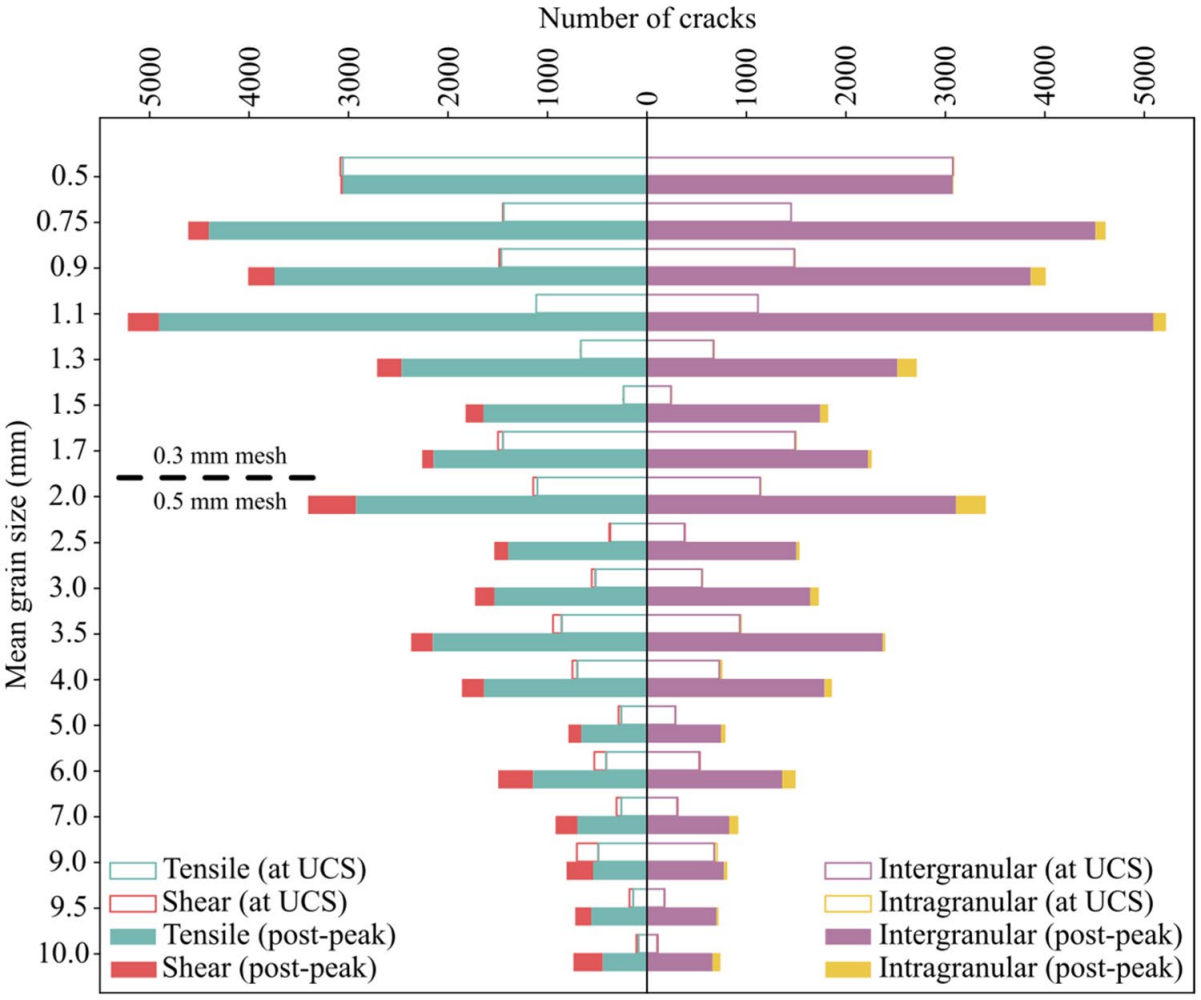
Number of intergranular, intragranular, shear, and tensile cracks for different mean grain size samples at UCS and 10% of strain in the post-peak. The total number of cracks shows a decreasing trend with coarsening of the grains in the samples. 0.5 mm mean grain size model was terminated at UCS. Models with average grain size ≤ 1.7 mm underwent mesh size refinement using 0.3 mm mesh size while 0.5 mm mesh was used for models with average grain size > 1.7 mm

**Fig. 19:**
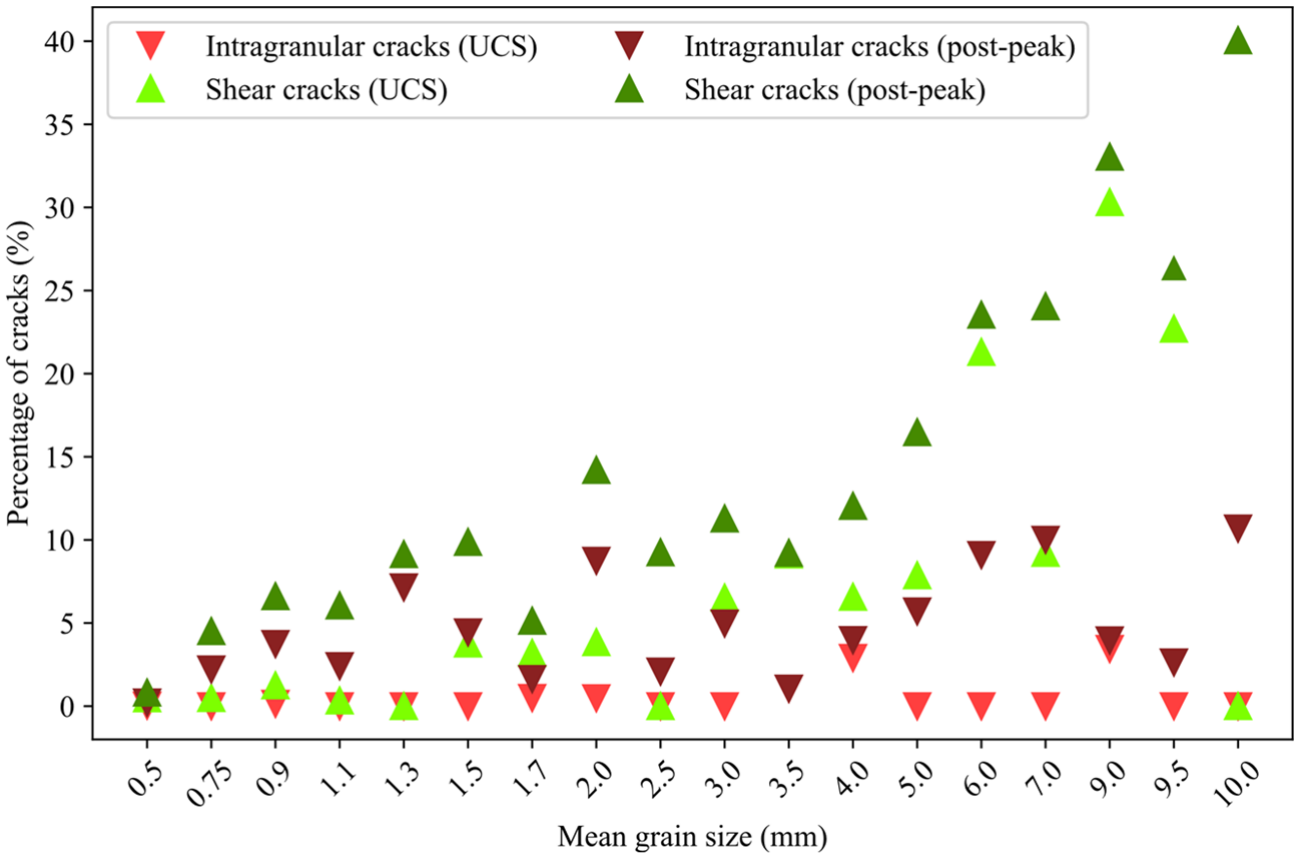
Percentage of intragranular and shear cracks for different mean grain size samples at UCS and 10% of strain in the post-peak

As can be seen from Fig. 18, the total number of cracks formed decreases with increasing grain size. Although intergranular tensile cracks were dominant in all the samples, a weak trend can be observed where the percentage of intragranular cracks increases in coarse-grained samples. This trend was clearer in the post-peak stage of the loading (Fig. 19). Shear crack percentages are higher in the simulated coarse-grained granites compared to fine-grained samples. The trend here is obvious at both peak (i.e., at UCS) and post-peak stages (Fig. 19).

**Fig. 20:**
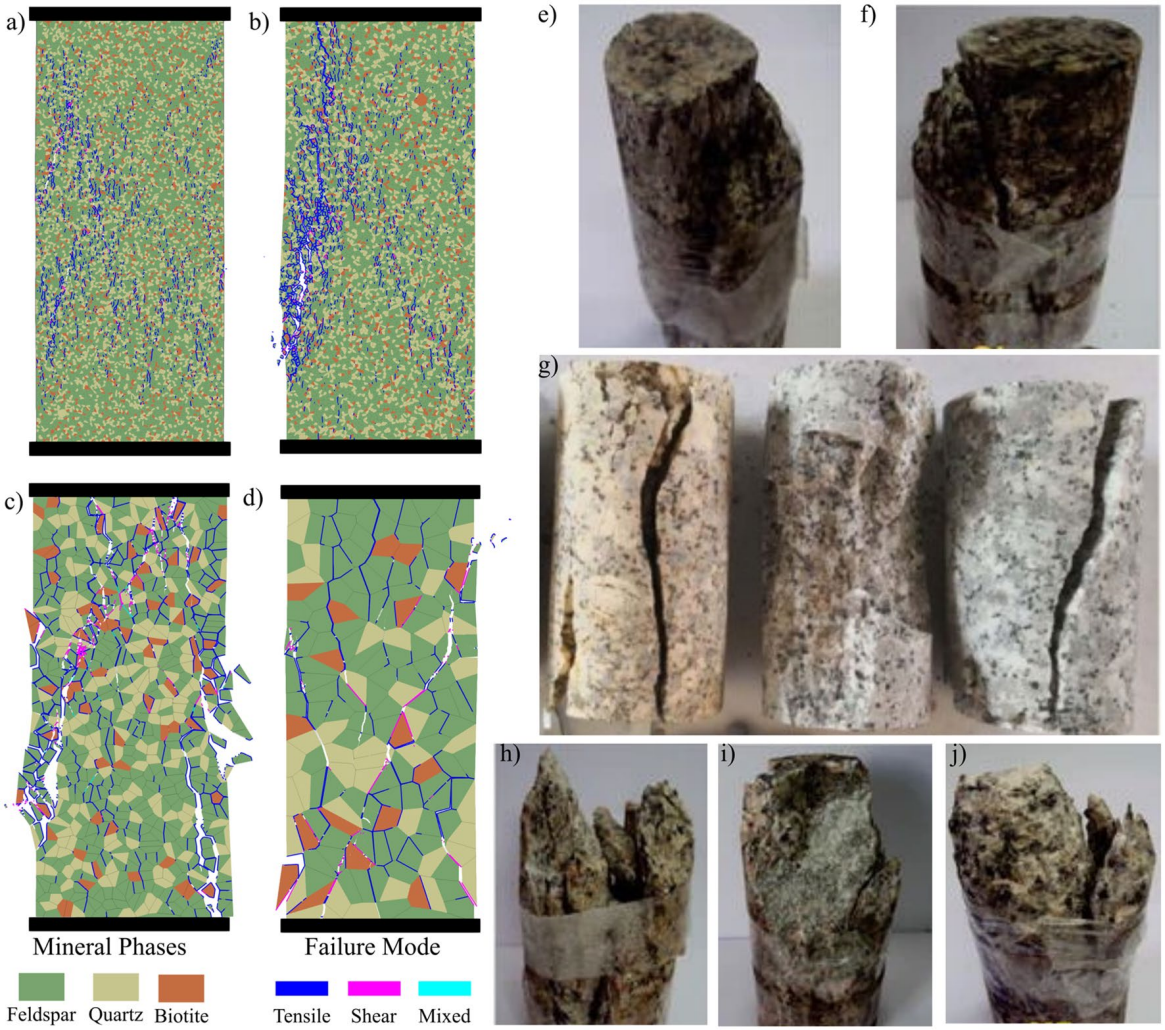
Macroscopic failure modes in different grain size models: **a** and **b** show shear zone formation in samples with mean grain sizes 0.9 mm and 1.1 mm, respectively. **c** Failing by multiple fracturing in 4.0-mm mean grain size sample. **d** axial splitting in 8.0 mm sample. Uniaxial laboratory testing of granitic rocks showed failure by shearing **e**–**f** (modified from Basu et al. (2013)), axial splitting **g** (modified from Xue et al. (2014)), and multiple fracturing **h**-**j** (modified from Basu et al. (2013))

Fine-grained samples tended to fail by localization of cracks in shear damaged zones promoted by crack coalescence associated with stress increase. These shear zones are inclined to the applied load (Fig. 20a and b). While in coarser grain samples, cracks were more distributed throughout the sample and their coalescence resulted in axial splitting parallel or subparallel to the applied load (Fig. 20c). In coarse-grained samples, cracks that initiated alongside long vertical or subvertical grain boundaries coalesced by forming wing cracks resulting in a vertical macroscopic crack (Fig. 20d).

### Effects of Mineralogical Composition on Mechanical Behavior

**Fig. 21:**
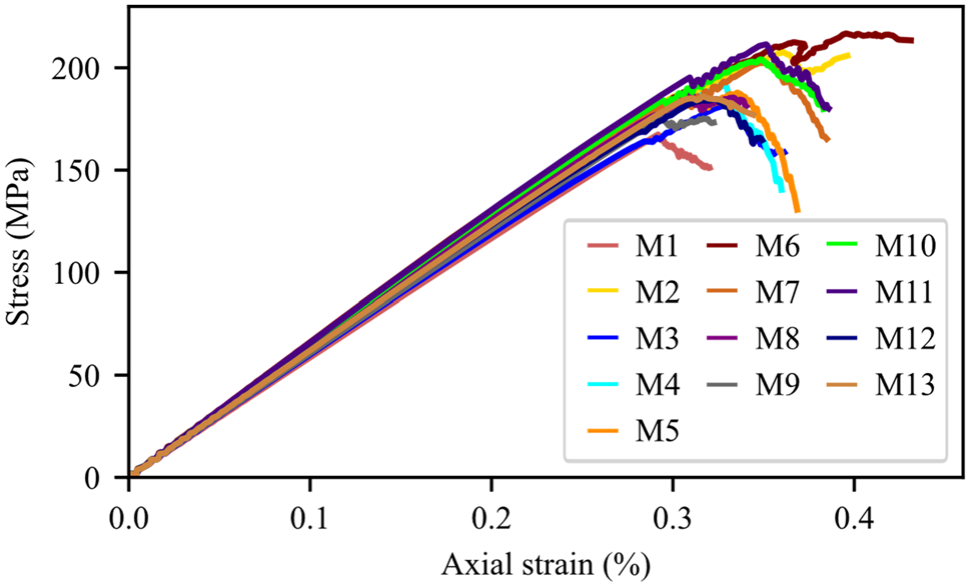
Complete stress–strain curves for synthetic granite samples with different mineralogical compositions (M1–M13 as listed in Table 5)

**Fig. 22:**
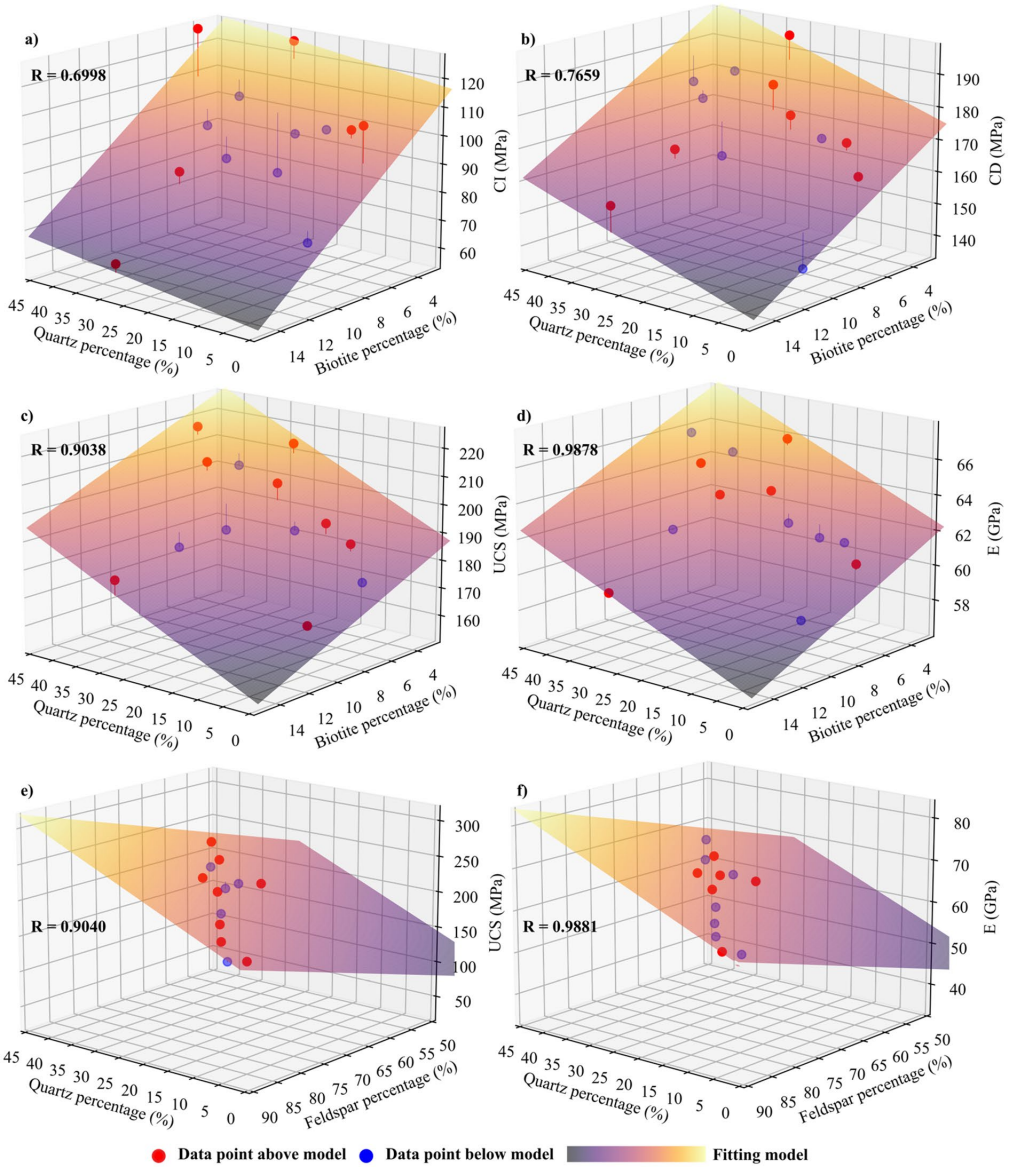
Multiple regression results. **a** CI, **b** CD, **c** UCS, and **d** E evolution as a function of quartz and biotite percentages. **e** UCS and **f** E evolution as a function of quartz and feldspar percentages

**Fig. 23:**
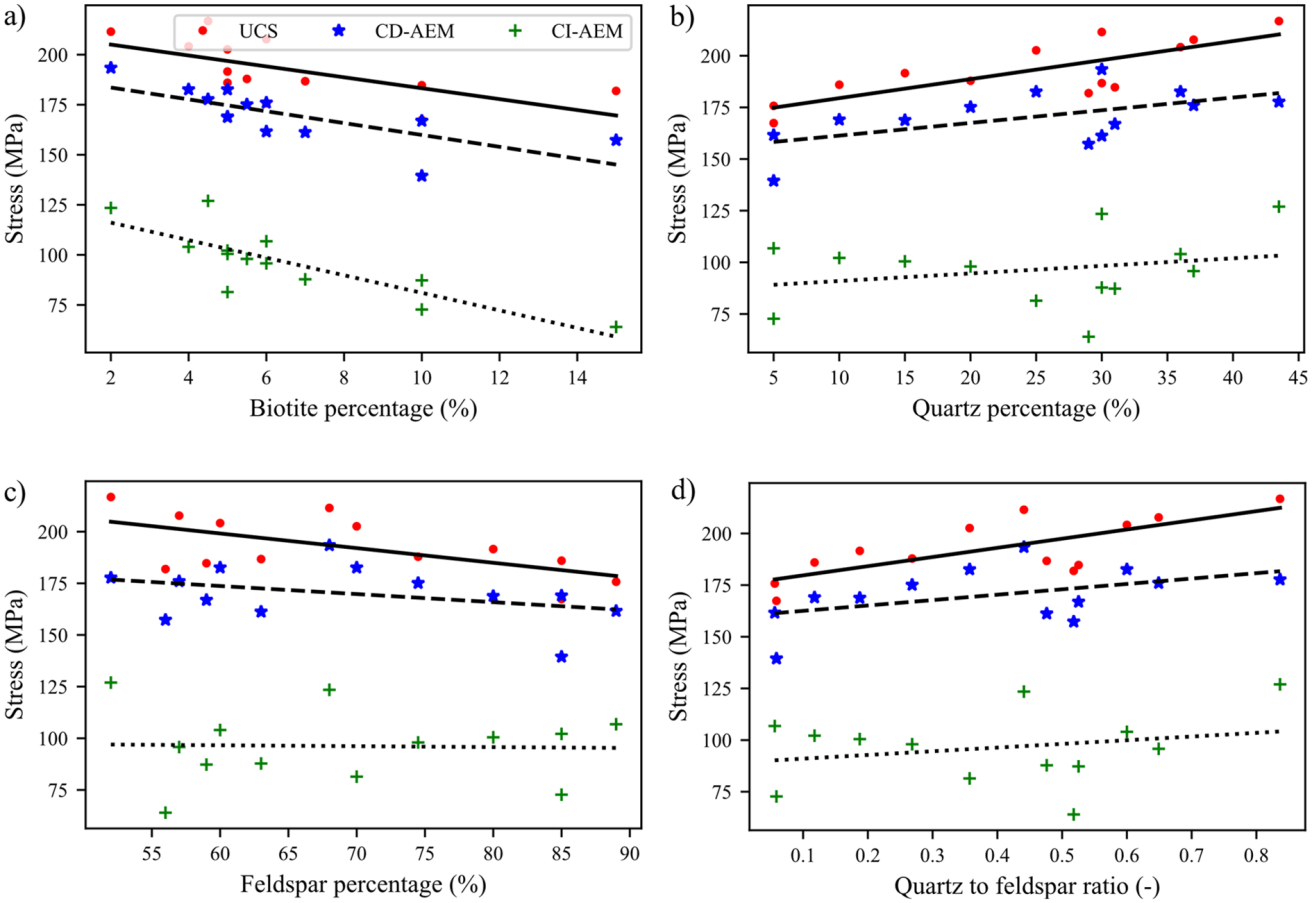
Peak strength and stress thresholds as a function of the relative amount of **a** biotite content, **b** quartz content, **c** feldspar content, and **d** quartz to feldspar ratio

Results of different mineralogy were analyzed to investigate the effect of biotite, quartz, and feldspar contents and quartz to feldspar ratio on the damage stress thresholds, UCS, and E. Complete stress–strain curves for the tested synthetic samples with different mineralogical compositions are shown in Fig. 21, and the influence of the mineral composition on rock deformation behavior is illustrated with multiple regression (Fig. 22) and simple regression (Fig. 23). Both UCS and E decreased with increase in biotite content (Figs. 22c and d and Fig. 23a). Noteworthy is the fact that for a given biotite content, the mechanical properties of samples, namely UCS and E, varied significantly depending on the quartz to feldspar ratio (Figs. 22 and 23a). Biotite content lowered the stress at which crack initiation, unstable growth, and coalescence occur (Fig. 22a and b). Moreover, improved mechanical properties in terms of CI, CD, UCS and E were associated with increased quartz content (Figs. 22 and 23b). Effect of feldspar content on mechanical properties is dependent on both quartz and biotite contents; increasing feldspar to quartz ratio resulted in mechanical properties deterioration (Fig. 22e and f); however, when the feldspar to biotite ratio increased, the mechanical properties improved. In Fig. 23d CI, CD, and UCS were plotted versus quartz to feldspar ratio. Increased quartz to feldspar ratio increased CI, CD, and UCS (Fig. 23d).

**Fig. 24:**
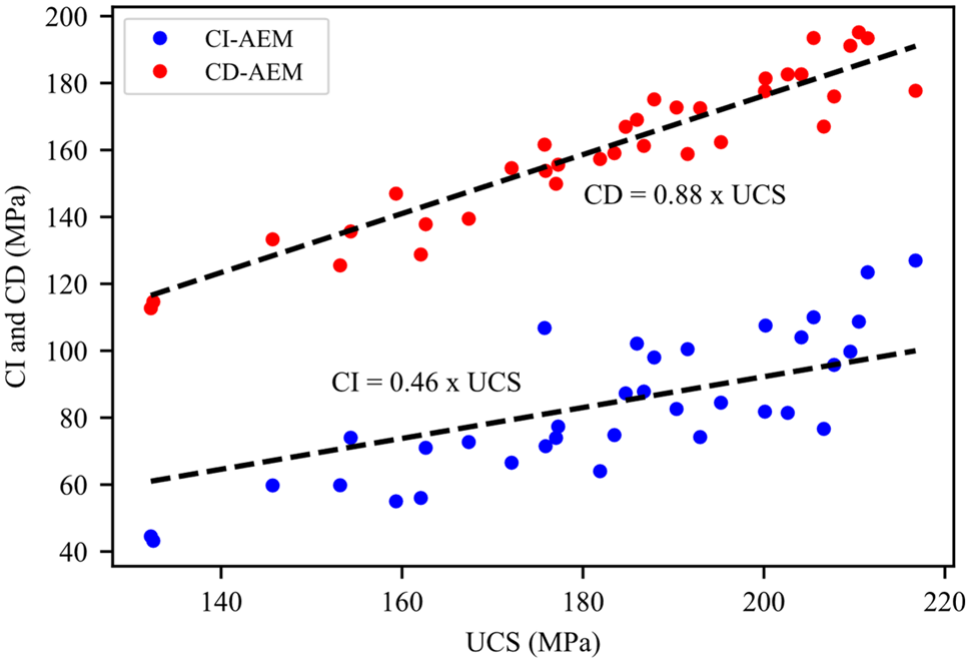
Crack initiation (CI) and crack damage (CD) stress thresholds versus peak strength values for all synthetic samples used in the study. Data fitting shows that CI and CD occur at around 46% and 88% of the peak strength (UCS), respectively

**Fig. 25:**
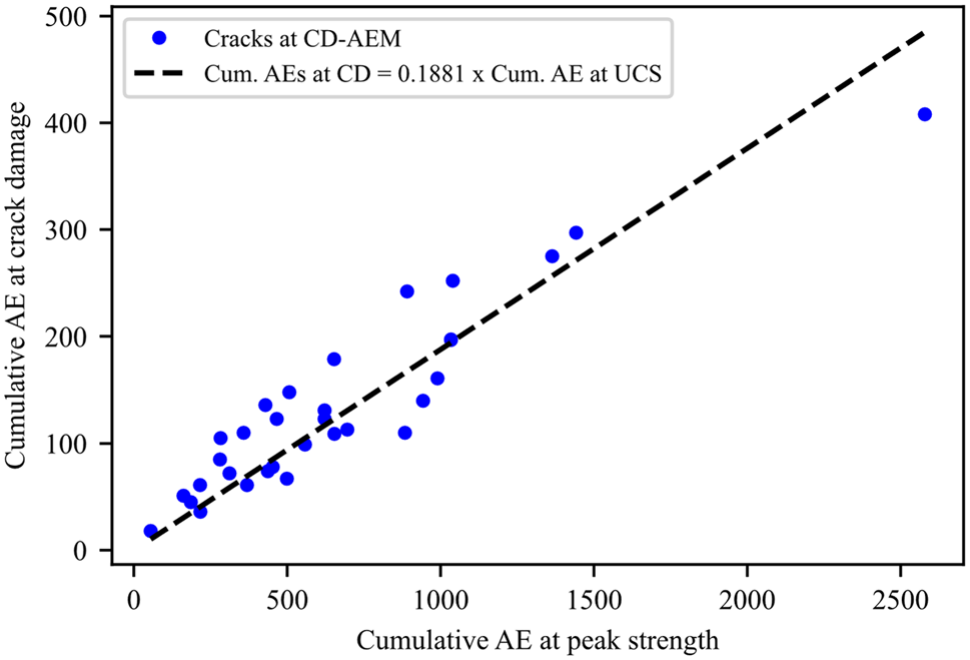
Cumulative AE events recorded at crack damage (CD) threshold versus AE recorded at peak strength (i.e., failure point)

By analyzing the data from all the synthetic samples tested in this study, CI ranges from 25 to 52% of the peak strength, with an average CI stress at 46% of the UCS (Fig. 24). The average CD of the tested samples was 88% of the UCS (Fig. 24). Moreover, the accumulated damage assessed by the percentage of cumulative AE at crack damage to that at the peak strength falls between 10 and 24%. Data fitting of recorded AE events at CD stress threshold versus cumulative AE events at peak strength for all tested samples shows a slope of 0.188 (Fig. 25).

## Discussion

**Fig. 26:**
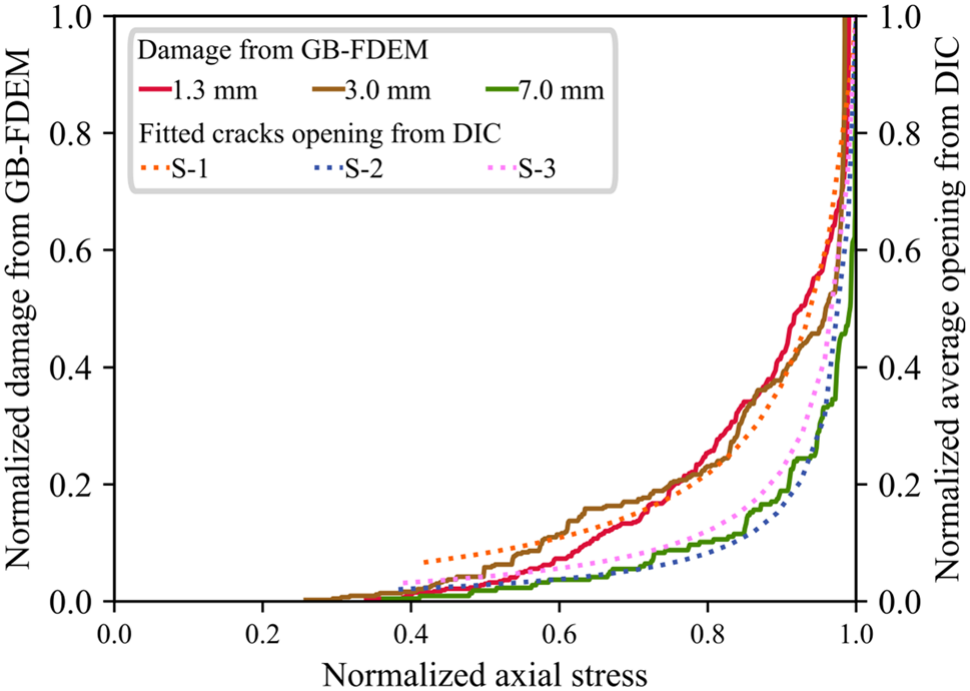
Normalized damage from GB-FDEM (solid lines) and normalized fitted cracks opening measurements from DIC (dotted lines) plotted versus normalized stress. Models with mean grain sizes of 1.3 mm, 3.0 mm, and 7.0 mm are shown as examples

In this paper, the evolution of the displacement field acquired by the DIC was used to infer crack damage thresholds and failure patterns in uniaxially compressed granite. In addition to clearly identifying the crack damage thresholds, this method provided useful information on the evolution of the crack opening and crack opening rate during the loading process in comparison to the commonly used methods for damage threshold identification such as strain and AE methods (Zhang et al. 2020). Crack opening provided information on the CI threshold range and the acceleration of the crack opening clearly marks the CD threshold. However, crack opening measurement inferred from DIC is highly dependent on the spatial resolution used in the DIC, and hence affects the CI threshold determination. In addition, using the DIC we were only able to detect surface cracks in the monitored area in contrast to AE which can capture cracking events across the bulk of the sample. To tackle these limitations, we supplemented DIC analysis with GB-FDEM simulation results for further discussion. We computed the normalized crack opening in the DIC analysis by calculating the ratio between the averaged opening of the six tracked macrocracks and the corresponding opening at the UCS. We also calculated the normalized damage from GB-FDEM using the ratio between the number of microcracks at each stress level to the number of microcracks at UCS. These two normalized values show clear similarities when examined as functions of the normalized stress (Fig. 26), and they increase gradually at a slow rate and transition into rapid increase after the CD threshold. Therefore, tracking crack opening using DIC and modeling using GB-FDEM can both be used to infer the damage conditions in the rock.

**Fig. 27:**
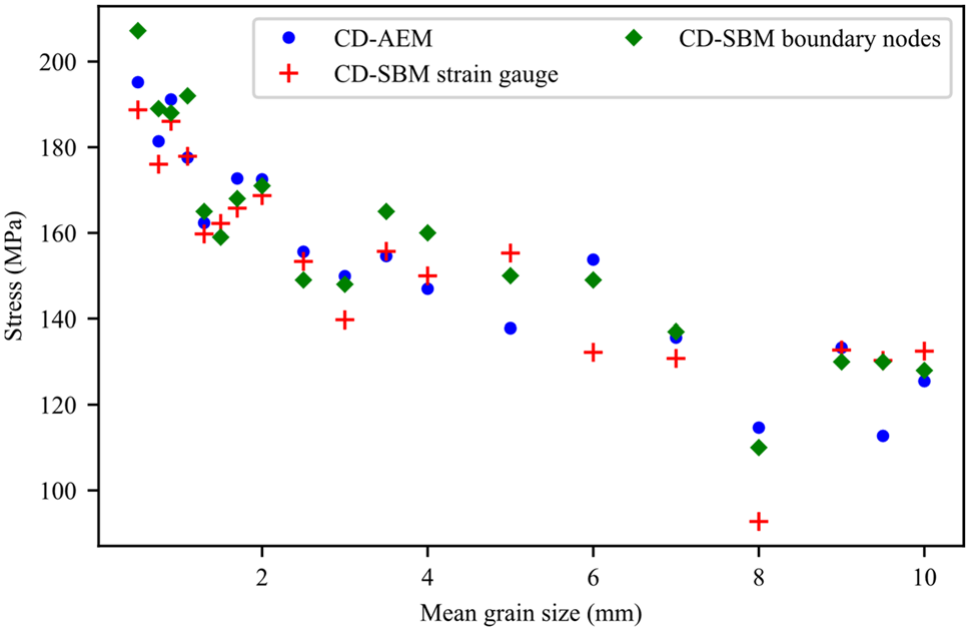
Crack damage thresholds inferred from AE data and volumetric strain using virtual strain gauge and sample boundary nodes tracing

**Fig. 28:**
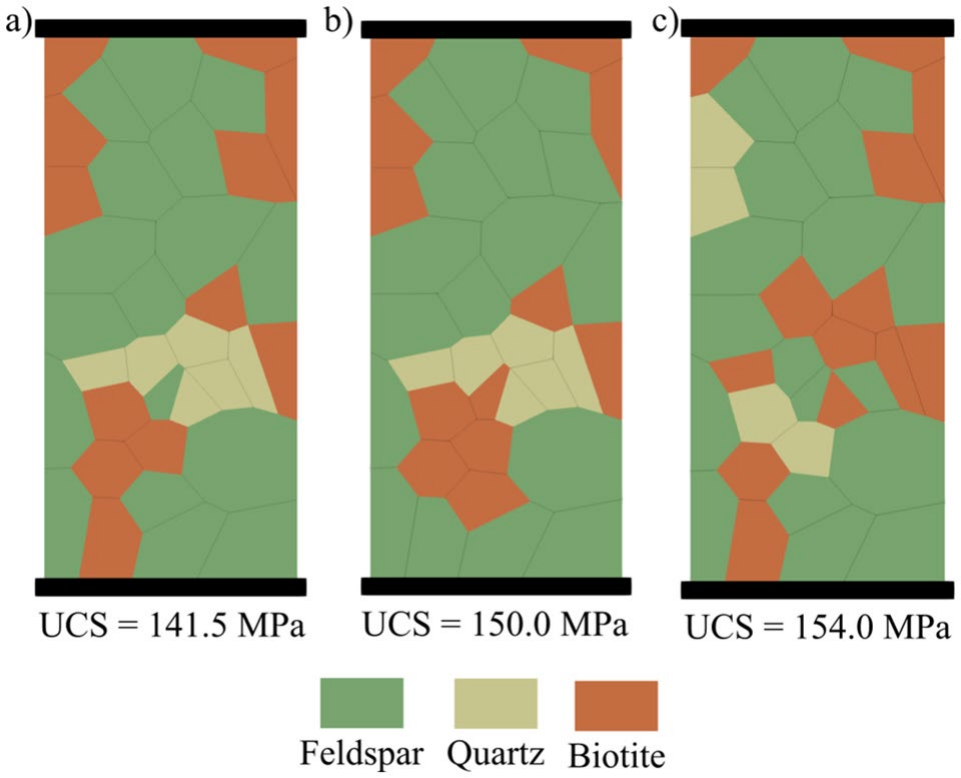
Effect of mineral grains arrangement on the UCS in pegmatite. Changes in UCS are within 9% of the calibration model

Using GB-FDEM simulations, both SBM and AEM were used to infer damage stress thresholds. It is important to note that in the SBM, the damage stress threshold values are highly biased by the calculation of the elastic parameters, especially Poisson’s ratio. However, such limitations can be overcome by adopting synthetic numerical models. Zhang et al. (2020) compared these methods and concluded that the AEM is the least uncertain. It is noteworthy that the 2D plane-strain FDEM simulations are not capable of capturing volumetric strain calculations. However, the areal strain calculated from the 2D simulations was used as a proxy for the volumetric strain. Our results show that the damage stress thresholds and UCS were affected by the grain size and data correlations show log-linear relations, consistent with experimental observations on granitic rocks under uniaxial compression (Eberhardt et al. 1999; Přikryl 2001). CI dependence on the grain size conforms with previous findings (Hatzor et al. 1997; Přikryl 2001) and agrees with observations on the medium-grained grey granite and the fine-grained granodiorite (Diederichs et al. 2004). Diederichs et al. (2004) correlated the higher crack initiation stress in granodiorite to its finer grains and less heterogeneous microstructure. However, correlations show that CI is less dependent on grain size compared to CD and UCS values. CD-SBM inferred from volumetric strain calculated by tracing the boundary nodes and from virtual strain gauges showed matching trends with CD-AEM values (Fig. 27). Moreover, this analysis suggests that not only grain size, but also the orientation of grain boundaries within the sample influence crack initiation, damage propagation, and ultimately peak strength. This effect was insignificant in fine-grained samples and became pronounced in coarse-grained samples. For instance, samples with 8 and 9 mm mean grain size exhibited lower strength than 10 mm mean grain size sample (Fig. 15). This was mainly due to the presence of more grain boundaries aligned sub-vertically in 8 and 9 mm mean grain size samples that facilitated crack initiation and coalescence; hence, promoting damage at lower stress levels. Moreover, in coarse-grained samples such as pegmatite, the spatial arrangement of the mineral grains affected the UCS values (Fig. 28), although this effect was limited to 9% of the UCS value for the model used in calibration.

**Fig. 29:**
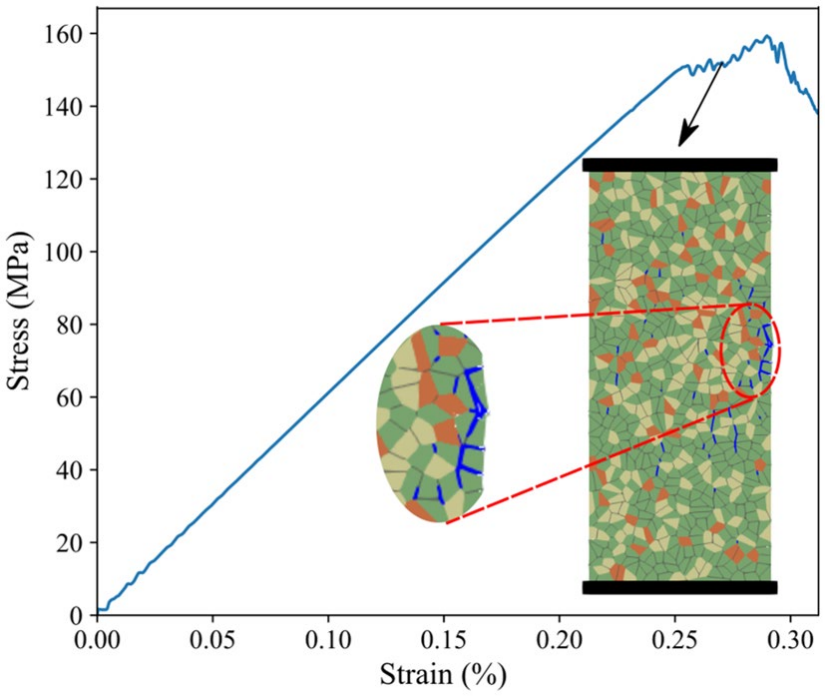
Complete stress–strain diagram for 4-mm mean grain size synthetic sample. Detachment of side grains (encircled in red) caused stress drops. With continued movement of the platens, a new force chain was formed allowing the sample to rebuild its strength

Complete stress–strain diagram of the coarse-grained samples (e.g., 4-mm and 9-mm samples in Fig. 17) exhibited double-peak behavior. This is due to the detachment of side grains which causes stress drops. With continued movement of platens, a new force chain was formed allowing the sample to rebuild its strength; when the second peak was reached (i.e., the peak strength), the synthetic sample abruptly failed (Fig. 29), resulting in a stepwise increase in the AE cumulative count evolution. The sudden increase in AE was attributed to the unstable propagation of cracks along large grain boundaries in these samples. This resembles the behavior of coal and conglomerate where the crack propagation occurs along large boundaries of aggregates, resulting in peaks of AE hits (Ding et al. 2019; Luo et al. 2021). Moreover, for the same mesh size, fine-grained samples experienced higher numbers of microcracking than coarse-grain samples (Fig. 18), conforming with experimental observations by Brooks (2013).

**Fig. 30:**
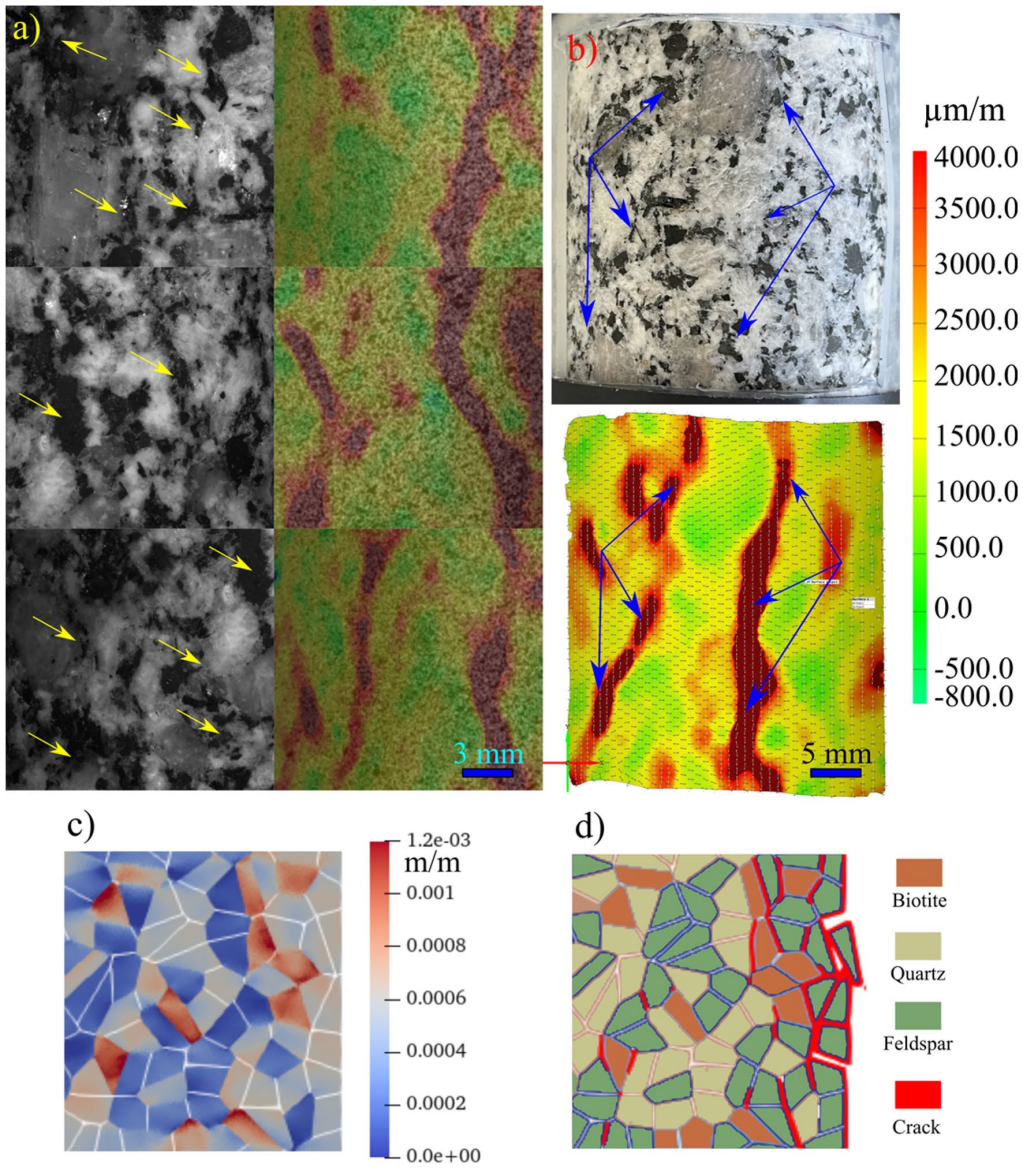
Strain localization and cracks nucleation along biotite-quartz/feldspar grain boundaries in samples **a** S-1 and **b** S-2, **c** high strain contrast between the biotite and other minerals in the model, and **d** cracks nucleate along biotite–quartz and biotite–feldspar grain boundaries from GB-FDEM simulations

Biotite content had a significant effect on the mechanical behavior even in a relatively small amount. Experimental work carried out on stress-induced microcracking in granite observed that biotite is a source of transgranular cracks in nearby quartz and feldspar (Ghasemi et al. 2020; Tapponnier and Brace 1976). DIC results revealed that biotite grains played a crucial role in nucleating cracks along the boundaries they shared with quartz and feldspar grains due to the stiffness contrast. This agrees well with the GB-FDEM simulations (Fig. 30). Feldspar, which is characterized by cleavage weakness planes, resulted in similar behavior, however, less severe as noticed from the slope of the trendline (Fig. 23). Data were fit by a linear curve that agrees with experimental data fitting in published literature (Tuğrul and Zarif 1999). In contrast, increased quartz content led to an increase in crack damage and UCS, and to a lower extent in the case of crack initiation, in agreement with published observations (Ghasemi et al. 2020). Furthermore, we did not observe any clear correlation between minerals content and the total number of cracks, or the mode of cracking. The complete stress–strain diagrams for the tested synthetic samples did not show any significant effect of grain size on E (Fig. 17) similar to previous numerical findings (Eberhardt 1998). However, it is evident that the mineralogical composition has a significant effect on E.

Our synthetic samples experienced increasing intragranular cracking and coalescence of these cracks into transgranular cracks, which agrees with microscopic observations from granitic rocks (Ghasemi et al. 2020; Lim et al. 2012) and numerical simulations (Li et al. 2021; Saadat and Taheri 2019a, b). DIC and GB-FDEM analysis revealed that tensile cracking was prevailing. The dominance of tensile cracking during uniaxial loading and the increase in shear cracking after the crack damage towards the post-peak stage were also documented both in Lac du Bonnet granite (Martin and Chandler 1994) and numerically (Li et al. 2021). The increasing intragranular cracking in coarse-grained samples is mainly due to the reduction in the weak interfaces represented by grains boundaries in coarse-grained samples; therefore, more transgranular cracks (captured as intragranular cracks) propagate through grains in samples with coarser grains. Shear cracks percentages are also higher in the simulated coarse-grained granites compared to fine-grained samples. Higher shear cracking percentages is due to the reduction of the grain boundaries which can result in cross-grain shear cracks formation.

Finally, CI and CD of the tested samples were in good agreement with experimental and numerical studies of crack initiation and damage stress thresholds (Li et al. 2020; Moradian et al. 2016; Zhang et al. 2020). Furthermore, around 18.8% of AE events were recorded up to CD and the remaining occurred towards the peak strength and post-peak stage. This is consistent with the experimental results of AE recordings (Eberhardt et al. 1999; Kallimogiannis et al. 2017; Kim et al. 2015). The linear correlation between the cumulative AE events at CD and UCS suggests the possibility of forecasting the failure point of a granitic rock by AE monitoring. In this work, we adopted a two-dimensional approach to model the effects of mean grain size and mineralogy on granite behavior; however, laboratory experiments involve three-dimensional phenomena. Therefore, AEs trends in the synthetic samples were compared to their experimental counterparts rather than the absolute counts. Moreover, the effects of grain size and mineralogy were inferred under uniaxial compression conditions. Future work will involve three-dimensional simulations of the granitic behavior under triaxial conditions to infer the limitations of the two-dimensional modeling approach.

## Conclusions

This paper adopted DIC and GB-FDEM techniques to infer insights on the evolution of damage stress thresholds, cracks opening, and failure patterns in granitic rocks. The influence of grain size and mineralogical composition on the mechanical parameters and damage stress thresholds of granitic rocks was investigated using GB-FDEM. Our models successfully captured the mechanical behavior of the three granitic rocks and their respective crack initiation (CI) and crack damage (CD) stress thresholds.

- DIC analysis of the uniaxially loaded samples showed that cracks preferentially nucleated along grain boundaries shared with biotite grains driven by the contrast in minerals stiffness. Crack initiation was inferred from the displacement field and indicated a CI threshold from 30.0% to 44.5% of the UCS value. Crack opening increased with the applied axial load and the opening rate increased after the CD threshold was reached (at 80.8–85% UCS).
- Both DIC and GB-FDEM results showed tensile dominant cracking with few shear cracks forming approaching the peak strength. In addition, crack opening evolution inferred from DIC correlates with the damage accumulation inferred from the AE count from the GB-FDEM simulations.
- GB-FDEM results exhibit a decrease in peak strength (UCS) values and damage stress thresholds with increasing grain size, following log-linear relationships. However, the CI threshold showed less dependency on the grain size than CD and peak strength, while grain size had no obvious effect on Young’s modulus of the rock. Moreover, the synthetic samples showed average crack initiation and crack damage stresses at 46% and 88% of the UCS, respectively, in good agreement with the experimental literature.
- Failure patterns were dictated by the grain size. Coarse-grained samples tended to fail by axial splitting, while macroscopic shear failures were typical for fine-grained samples. Interestingly, for the same mesh size, coarse-grained samples underwent less microcracking compared to fine-grained samples, which agrees well with experimental literature on fracture process zone development in fine- and coarse-grained granites.
- Higher biotite content caused deterioration of the mechanical properties, while higher quartz content had a much more favorable impact. Similar impacts were also observed on the crack damage stress thresholds, UCS, and E, which showed favorable impacts to increased quartz to feldspar ratio.

This work highlights the key role that grain size and mineralogical composition play in determining the mechanical response of the rock. This is particularly crucial in assessing the rock mass strength in drilling, excavation, and tunneling in brittle rock formations. Such assessment revolves around the crack initiation and crack damage thresholds, which aids in predicting failures of the rock mass. In this study, our proposed GB-FDEM model is rigorous enough to numerically identify CI and CD that correlate to the strength degradation in rock masses. The new insights also draw attention to the fact that microstructural effects of the rock are often overlooked in rock mass strength assessment. As such, the proposed approach can be further adopted to infer the microstructural features from AE by investigating the signature of microstructure on AE parameters such as b-value and emitted energy.

### Supplementary Information

Below is the link to the electronic supplementary material.

Supplementary file1 (PDF 584 KB)

## Data Availability

Data used in this manuscript are presented in tables and figures. Raw simulation files will be made available upon request.
