# Supplementary material for: Evaluation of Damage Stress Thresholds and Mechanical Properties of Granite: New Insights from Digital Image Correlation and GB-FDEM
Source: Rock Mech Rock Eng. 2024 Mar 6;57(7):4679–706. doi: 10.1007/s00603-024-03789-7 (PMC11236892; doi:10.1007/s00603-024-03789-7)
Supplement: Supplementary file 1 — Supplementary file1 (PDF 584 KB) [file 603_2024_3789_MOESM1_ESM.pdf]

Supplementary materials for

Evaluation of Damage Stress Thresholds and Mechanical Properties of Granite: New Insights from Digital Image Correlation and GB-FDEM

Kareem Ramzy Aboayanah[1], Aly Abdelaziz[1], Bezawit Fekadu Haile[1], Qi Zhao*[1,2], and Giovanni Grasselli[1]

[1]Department of Civil and Mineral Engineering, University of Toronto, 35 St George St., Toronto, ON M5S 1A4, Canada

[2]Department of Civil and Environmental Engineering, The Hong Kong Polytechnic University, Hung Hom, Hong Kong SAR, China.

*Corresponding author: Qi Zhao (qi.qz.zhao@polyu.edu.hk)

Major principal strain field for the samples S-2, S-3, and S-4:

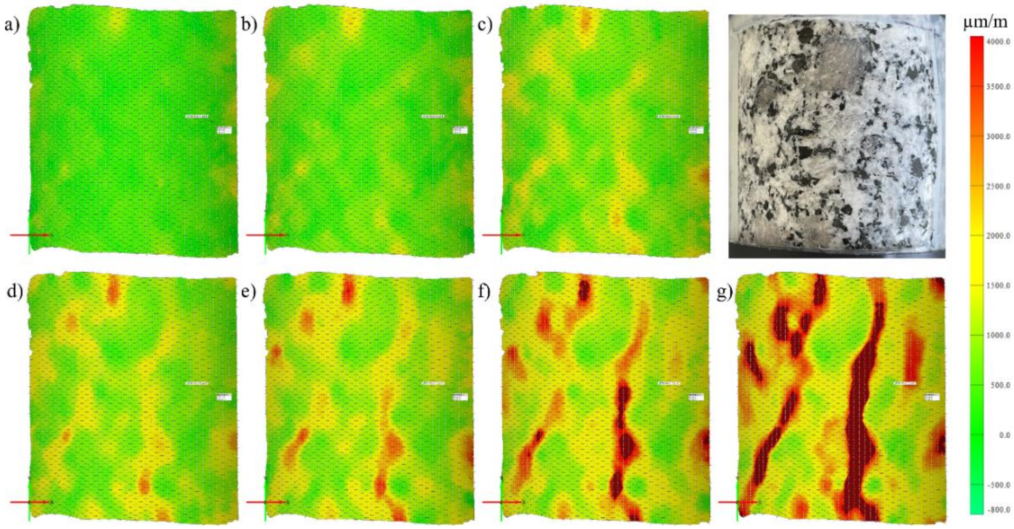

Figure S 1- Major principal strain field in sample S-2 at (a) 44.5, (b) 60, (c) 70, (d) 77.5, (e) 84, (f) 95, and (g) 100% UCS.

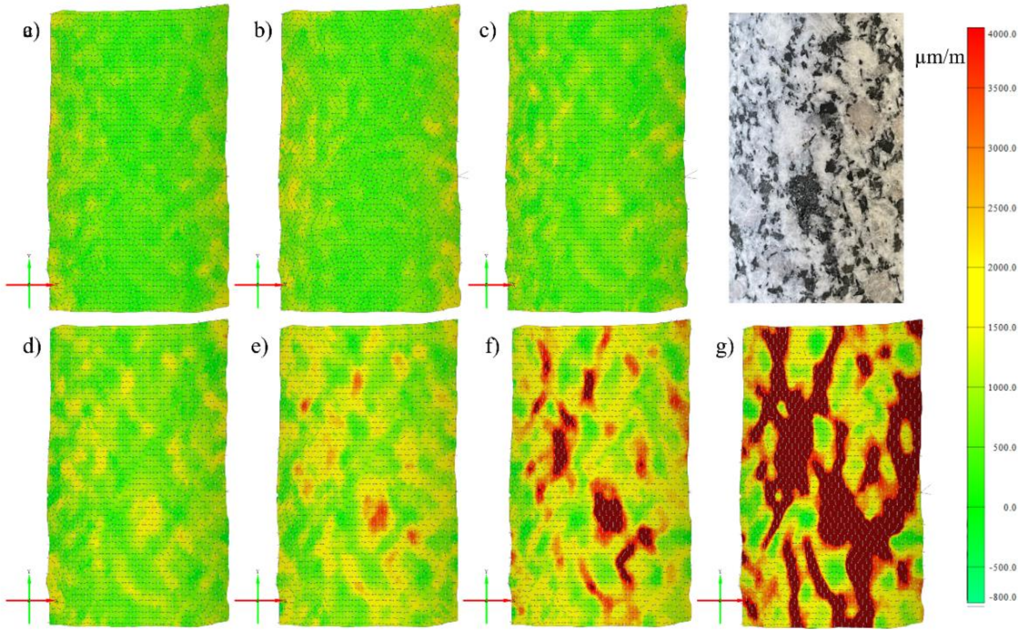

Figure S 2 - Major principal strain field in sample S-3 at (a) 44.5, (b) 60, (c) 70, (d) 77.5, (e) 84, (f) 95, and (g) 100% UCS.

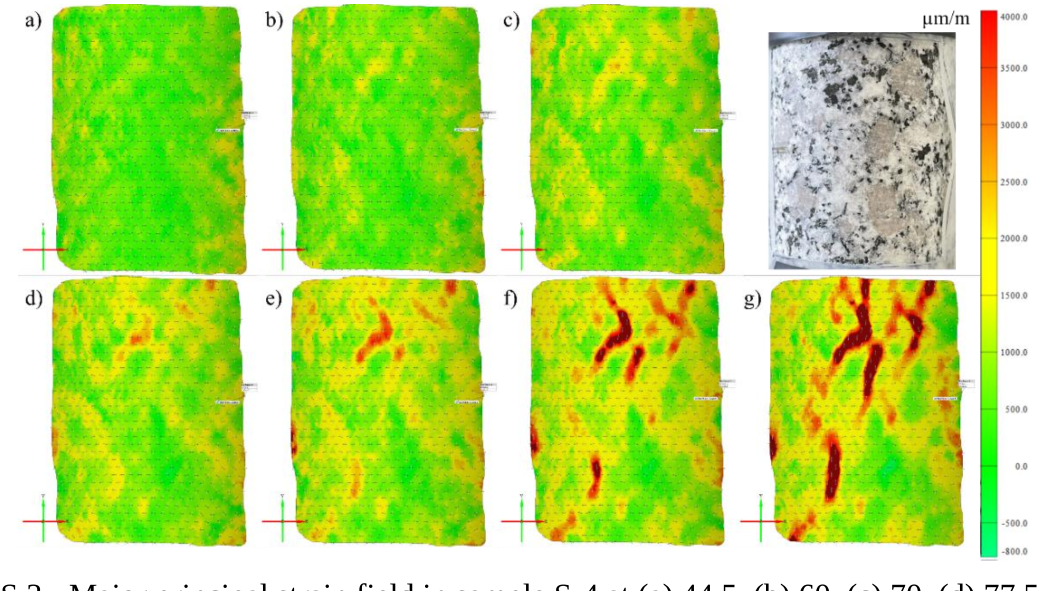

Figure S 3 - Major principal strain field in sample S-4 at (a) 44.5, (b) 60, (c) 70, (d) 77.5, (e) 84, (f) 95, and (g) 100% UCS.
